# New 3-Hydroxyquinaldic Acid Derivatives from Cultures of the Marine Derived Actinomycete *Streptomyces cyaneofuscatus* M-157

**DOI:** 10.3390/md16100371

**Published:** 2018-10-08

**Authors:** Francisco Javier Ortiz-López, Elsa Alcalde, Aida Sarmiento-Vizcaíno, Caridad Díaz, Bastien Cautain, Luis A. García, Gloria Blanco, Fernando Reyes

**Affiliations:** 1 Fundación MEDINA, Centro de Excelencia en Investigación de Medicamentos Innovadores en Andalucía, Avda. del Conocimiento 3, Parque Tecnológico de Ciencias de la Salud, E-18016 Granada, Spain; javier.ortiz@medinaandalucia.es (F.J.O.-L.); elsaalcalde@uma.es (E.A.); caridad.diaz@medinaandalucia.es (C.D.); bastien.cautain@medinaandalucia.es (B.C.); 2 Departamento de Biología Funcional, Área de Microbiología, and Instituto Universitario de Oncología del Principado de Asturias, Universidad de Oviedo, 33006 Oviedo, Spain; UO209983@uniovi.es; 3 Departamento de Ingeniería Química y Tecnología del Medio Ambiente, Área de Ingeniería Química, Universidad de Oviedo, 33006 Oviedo, Spain; luisag@uniovi.es

**Keywords:** 3-hydroxyquinaldic acid, *Streptomyces cyaneofuscatus*, deep-sea actinobacteria, Cantabrian Sea

## Abstract

Fractionation of the bioactive extract of a culture of the marine derived actinomycete *Streptomyces cyaneofuscatus* M-157 led to the isolation of the known 3-hydroxyquinaldic acid (**4**), its amide (**5**) and three new derivatives (**1**–**3**) containing different amino acid residues. The structures of the new molecules (**1**–**3**), including their absolute configuration, were determined by the analysis of their ESI-TOF MS and one-dimensional (1D) and two-dimensional (2D) NMR spectra and advanced Marfey’s analysis of their hydrolyzation products. Compound **3** spontaneously dimerized in solution to give the disulfide derivative **6**. Unfortunately, none of the new compounds isolated confirmed the antimicrobial activity found in the bacterial extract, perhaps indicating that such antibacterial activity might be due to presence in the extract at the trace level of larger bioactive 3-hydroxyquinaldic acid derivatives from which compounds **1**–**3** are biosynthetic precursors. Cytotoxicity tests confirmed the moderate and weak IC_50_ values of 15.6 and 51.5 µM for compounds **5** and **1**, respectively.

## 1. Introduction

Marine actinomycetes continue to be the source of new natural products with interesting bioactive properties. Among them, depsipeptides containing quinaldic acid moieties in their structures, also called chromodepsipeptides, constitute an interesting structural class due to their relevant biological activities. These include antibiotics, such as the antibiotics UK-63,052, UK-63,598, and UK-65,662 [1], and their sulfoxide derivatives RK-1355A and B [2], luzopeptins A–C [3,4,5], quinaldopeptin [6] and sandramycin [7,8], or potent cytotoxic agents, such as thiocoraline [9,10] or the structurally related thiochondrilline C [11]. Most of these compounds act as DNA bisintercalators, and in the case of thiocoraline, the two 3-hydroxyquinaldic moieties present in the molecule seem to confer stability to the complex with DNA as part of its mode of action [12].

A recent survey of marine actinomycete strains that were isolated from deep-sea water and invertebrates (1500–4700 m depth) collected during a cruise expedition to the submarine Avilés Canyon, Cantabrian Sea (Biscay Bay, Northeast Atlantic), identified 18 strains having antibiotic properties against a panel of Gram positive and Gram negative bacteria, including clinical isolates [13]. New natural products with antibiotic and cytotoxic activities were recently uncovered in diverse Actinobacteria that were isolated from this canyon [14,15,16]. We report here the discovery of a family of new compounds from one of these strains, M-157, isolated from a solitary coral belonging to the order Scleractinia collected at 2000 m depth in this Canyon, identified as *Streptomyces cyaneofuscatus* by 16S rDNA. LC-HRMS dereplication [17] of the antimicrobial ethyl acetate extract of this strain identified the presence of 3-hydroquinaldic acid together with some new structurally related natural products. Fractionation of this extract using reversed phase flash chromatography and HPLC yielded 3-hydroxyquinaldic acid, its amide and three new derivatives (**1**–**3**) containing different amino acids as the major components of this extract.

## 2. Results

### 2.1. Taxonomy and Phylogenetic Analysis of the Strain M-157

**Figure 1:**
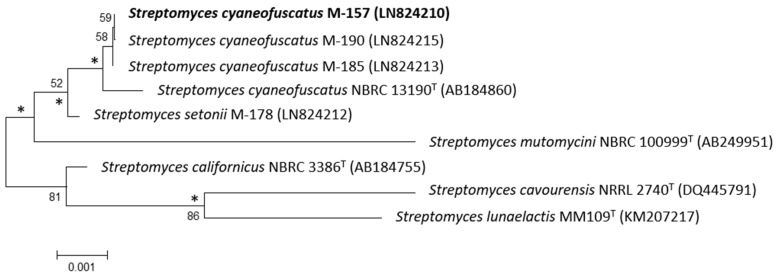
Neighbour-joining phylogenetic tree obtained by distance matrix analysis of 16S rDNA sequences, showing *Streptomyces cyaneofuscatus* M-157 position and most closely related phylogenetic neighbours. Numbers on branch nodes are bootstrap values (1000 resamplings; only values >50% are given). Asterisks indicate that the corresponding nodes were also recovered in the maximum likelihood tree. Bar indicates 0.1% sequence divergence.

The 16S rDNA of strain M-157 was previously amplified by polymerase chain reaction (PCR) and sequenced [13]. After Basic Logic Alignment Search Tool (BLAST) sequence comparison, strain M-157 showed 100% identity to *S. cyaneofuscatus* (Accession number NR_115383); thus, this strain was designated as *S. cyaneofuscatus* M-157 (EMBL Sequence number LN824210). The phylogenetic tree that was generated by neighbour-joining and maximum likelihood method based on 16S rDNA sequence clearly revealed the evolutionary relationship of strain M-157 with a group of known *Streptomyces* species (Figure 1).

### 2.2. Isolation and Structural Elucidation of Compounds ***1**–**3***

**Figure 2:**
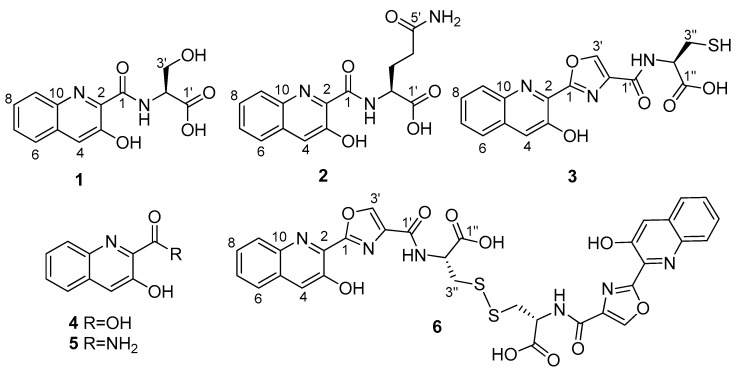
Compounds isolated from *Streptomyces cyaneofuscatus* M-157.

*S. cyaneofuscatus* M-157 was cultured in solid R5A medium and extracted with acidified ethyl acetate. This extract was fractionated using reversed phase C18 column chromatography followed by preparative HPLC to yield compounds **1**–**3**, together with the known compounds 3-hydroxiquinaldic acid (**4**) and its amide (**5**) (Figure 2), which were identified by ESI-TOF MS and comparison of their NMR spectra with those that were described in the literature [18,19]. During the structural characterization of compound **3**, a spontaneous dimerization through disulphide bond formation in DMSO-*d_6_* solution led to its full conversion into compound **6**.

**Figure 3:**
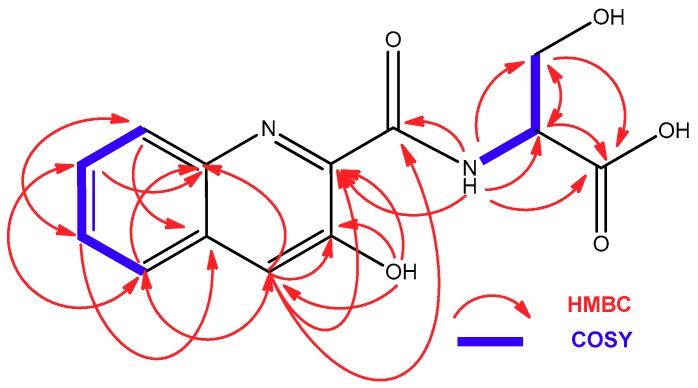
COSY and HMBC correlations observed in the structure of **1**.

Compound **1**, isolated as a yellow amorphous solid, possess a molecular formula of C_13_H_12_N_2_O_5_, deduced from the presence of a protonated ion at *m*/*z* 277.0821 in its ESI-TOF mass spectrum and 13 signals in its ^13^C NMR spectrum. Absorption maxima at 232, 308, and 360 nm in its UV spectrum and signals present in the aromatic region of its ^1^H (δ_H_ 8.05 d, 7.91 dd, 7.89 s, 7.66 m, and 7.63 m) and ^13^C NMR spectra (δ_C_ 153.1, 140.8, 134.6, 131.6, 129.0, 128.9, 127.8, and 126.7), together with the conjugated carbonyl signal in the latter spectrum at 167.8 ppm and a phenol group at δ_H_ 12.01 ppm confirmed the presence of a 3-hydroxyquinaldic moiety. The remaining signals observed in its ^1^H and ^13^C NMR spectra (Table 1) accounted for a nitrogenated methine (δ_H_ 4.61 ddd, δ_C_ 54.6), an oxygenated methylene (δ_H_ 3.98 dd and 3.89 dd, δ_C_ 60.8), a carbonyl group (δ_C_ 171.1), an amide NH group (δ_H_ 9.19 d), and one hydroxy group (δ_H_ 13.11 br s), the latter probably corresponding to a carboxylic acid functionality. COSY and HMBC correlations (Figure 3) assigned these signals to the presence of a serine residue that was linked to the 3-hydroxyquinaldic moiety through an amide bond between the amino group of the serine and the carbonyl group of 3-hydroxyquinaldic acid, as evidenced by HMBC correlations from the amide NH proton and H-1′ to the carbonyl carbon C-1. Advanced Marfey’s analysis [20] established the absolute configuration of the serine residue as L after hydrolysis, derivatization with *N*^α^-(2,4-dinitro-5-fluorophenyl)-l-valinamide (l-FDVA) and LC/MS analysis.

**Figure 4:**
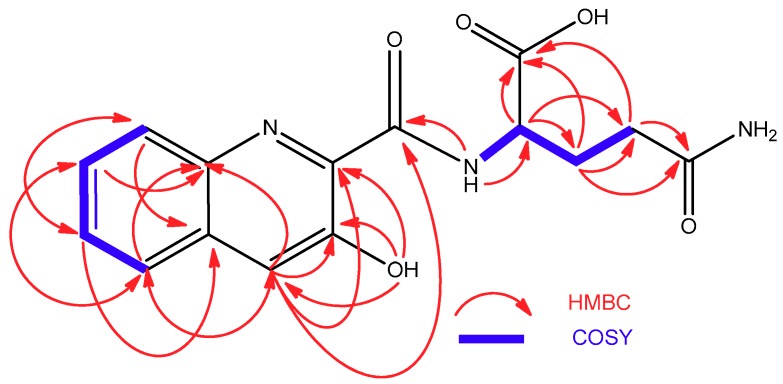
COSY and HMBC correlations observed in the structure of **2**.

The molecular formula of compound **2** was established as C_15_H_15_N_3_O_5_, according to the protonated ion observed at *m*/*z* 318.1088 in its ESI-TOF spectrum. Signals with similar chemical shifts to those of **1** in the aromatic regions of its ^1^H and ^13^C NMR spectra and UV absorption maxima at 232, 308, and 360 nm accounted again for the presence of a 3-hydroxyquinaldic moiety. The remaining signals that were observed in its NMR spectra were assigned to one nitrogenated methine (δ_H_ 4.53 ddd, δ_C_ 52.3), two aliphatic methylenes (δ_H_ 2.22 m (2H), δ_C_ 31.6, and δ_H_ 2.22 m and 2.11 m, δ_C_ 26.5), two carbonyl groups (δ_C_ 173.8 and 172.7), and amide NH (δ_H_ 9.53 d) and NH_2_ groups (δ_H_ 7.32 br s and 6.80 br s), which together with correlations observed in the COSY and HMBC spectra (Figure 4) indicated that they belonged to a glutamine (Gln) unit. The structure of compound **2** was finally established as the amide formed between the amino group of Gln and the carboxylate of 3-hydroxyquinaldic acid through the HMBC correlation observed between the amide NH proton and the carbonyl carbon C-1 (Figure 4). Hydrolysis in 2 N HCl followed by derivatization with *N*^α^-(2,4-dinitro-5-fluorophenyl)-l-valinamide (l-FDVA) and LC/MS analysis established the absolute configuration of the Gln residue present in **2** as l by comparison with glutamic acid standards.

**Figure 5:**
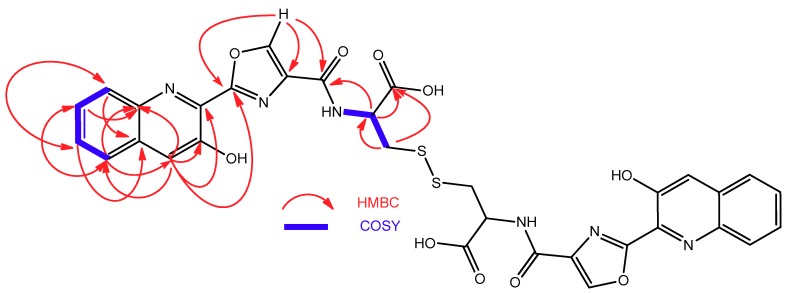
COSY and HMBC correlations observed in the structure of **6**.

ESI-TOF analysis of compound **3** rendered a molecular formula of C_16_H_13_N_3_O_5_S according to the protonated ion at *m*/*z* 360.0650 observed in its spectrum. In addition to the presence of a 3-hydroxiquinaldic acid moiety, evidenced by its UV spectrum and NMR signals with chemical shifts similar to those observed in **1** and **2**, the NMR spectra of **3** (Table 2) displayed signals indicative of the presence of a 2-substituted oxazole-4-carboxylate (δ_H_ 8.96 s, δ_C_ 159.2, 159.1, 143.6, and 136.2) and a cysteine residue [δ_C_ 172.0 (CO); δ_H_ 5.58 m, δ_C_ 55.3 (CH); δ_H_ 3.08 and 2.96 m, δ_C_ 25.6 (CH_2_); δ_H_ 9.20 (NH)]. Spontaneous conversion of **3** into its dimer **6** was observed in DMSO-*d_6_* solutions during 2D-NMR acquisition. After complete conversion, the presence of **6** was evidenced by changes in the cysteine ^1^H and ^13^C NMR chemical shifts (Table 2), particularly those of the methylene C-3′’ attached to the sulfur atom, whose ^13^C signal was highly deshielded from 25.6 in **3** to 39.3 ppm in **6** due to the formation of the disulfide bridge. COSY and HMBC correlations (Figure 5) that were observed in the spectra of **6** established the structures of both compounds, the monomer and the dimer, as depicted in Figure 2. This full conversion was confirmed by ESI-TOF analysis of the recovered sample, where a protonated ion observed at *m*/*z* 717.1057, accounting for a molecular formula of C_32_H_25_N_6_O_10_S_2,_ was the unique component detected. This spontaneous dimerization was further monitored by time-course ^1^H NMR experiments with a fresh sample of monomer **3** (see Figure S32). Finally, the absolute configuration of the cysteine residue in compound **3** (and therefore that of the dimeric compound **6**) was determined to be l by Marfey’s analysis of the corresponding cysteic acid derivative [21]. For this purpose, compound **3** was oxidized with performic acid and the resulting product was hydrolyzed, subsequently derivatized with l-FDVA and then analyzed by LCMS. Derivatization in parallel of a standard of l-cysteic acid derivatized with both, l and d-FDVA, provided the l-cysteic-l-FDVA and l-cysteic-d-FDVA (enantiomer of d-cysteic-l-FDVA) standards that are necessary to perform the Marfey’s analysis. 

Compounds **1**, **2** and **4**–**6** were tested against a small panel of pathogenic bacteria, including one Gram positive (methicillin resistant *Staphylococcus aureus*, MRSA) and two Gram negative (*Escherichia coli* and *Acinetobacter baumannii*) strains. None of the compounds displayed antimicrobial activity at the highest concentration tested of 64 µg/mL. Additionally, cytotoxicity tests against the human tumor cell line HepG2 were also performed. Moderate and weak activities were observed for compounds **5** and **1**, with IC_50_ values of 15.6 and 51.5 µM, respectively. No activity was observed for the rest of the compounds at the highest concentration tested (20 µg/mL).

## 3. Discussion

Three new 3-hydroxyquinaldic acid derivatives were isolated from the bioactive extract of *S. cyaneofuscatus* M-157. The structure of all the molecules isolated, including their absolute configurations, were fully established by HRMS, NMR, and Marfey’s analysis. Compound **3** is most probably biosynthesized from the serine precursor **1** via elongation of the peptidic sequence with a cysteine residue and post-translational heterocyclization (cylodehydration and dehydrogenation) of the serine residue onto the carbonyl group of quinaldic acid to form an oxazole ring [22]. Most of the compounds isolated were tested against a set of pathogenic microorganisms, but none of them displayed the antimicrobial properties that are found in the crude extract. Additional tests against the human tumor cell line HepG2 were also performed, indicating moderate and weak activity for compounds **5** and **1**, respectively. Other bioactive compounds with an elongated peptide chain might also be present in trace amounts in the extract. Interestingly, the three new compounds isolated possess structural features that are compatible with being biosynthetic precursors of larger chromodepsipeptides, similar to known antibacterial or cytotoxic agents, such as the luzopeptins, quinaldomycin, sandramycin, or thiochoraline, whose presence in low amounts in the extract would explain the antimicrobial properties that were observed.

To prove this hypothesis, experimental evidences of the production of such molecules should be obtained, perhaps using cultivation-based techniques (OSMAC approaches) to try to maximize the number of different molecules produced by our microbial strain and eventually trigger the production in higher amounts of the actual bioactive metabolites [23].

## 4. Materials and Methods

### 4.1. General Experimental Procedures

Optical rotations were measured using a Jasco P-2000 polarimeter (JASCO Corporation, Tokyo, Japan). UV spectra were obtained with an Agilent 1100 DAD (Agilent Technologies, Santa Clara, CA, USA). IR spectra were recorded on a JASCO FT/IR-4100 spectrometer (JASCO Corporation, Tokyo, Japan) equipped with a PIKE MIRacle^TM^ single reflection ATR accessory (PIKE Thecnologies Inc., Madison, WI, USA). NMR spectra were recorded on a Bruker Avance III spectrometer (500 and 125 MHz for ^1^H and ^13^C NMR, respectively) equipped with a 1.7 mm TCI MicroCryoProbe^TM^ (Bruker Biospin, Falländen, Switzerland). Chemical shifts were reported in ppm while using the signals of the residual solvent as internal reference (δ_H_ 2.50 and δ_C_ 39.5 for DMSO-*d*_6_). LC–MS and LC–HRMS analyses were performed, as described previously [24].

### 4.2. Taxonomic Identification of the Producing Microorganism

Strain *S. cyaneofuscatus* M-157 was subjected to phylogenetic analysis based on 16S rDNA sequence analysis [13]. Phylogenetic analysis was performed using MEGA version 6.0 [25] after multiple alignment of data by Clustal Omega [26]. Distances (distance options according to the Kimura two-parameter model [27]) and clustering with the neighbour-joining [28] and maximum likelihood [29] methods were evaluated using bootstrap values that were based on 1000 replications [30].

### 4.3. Fermentation of the Producing Microorganism

100 petri dishes with R5A solid medium [31] were inoculated with spores of strain M-157. After three weeks at 28 °C, plates were extracted with ethyl acetate under acidic conditions (1% formic acid). The extract was evaporated to dryness, resuspended in tert-butanol: water (1:1) and freeze dried.

### 4.4. Extraction and Bioassay-Guided Isolation

The whole biomass resulting from a 100 Petri dishes culture of strain M-157 was extracted with ethyl acetate under acidic conditions (1% formic acid). The extract was evaporated to dryness, resuspended in *tert*-butanol: water (1:1) and freeze dried. The lyophilized powder (ca. 600 mg) was disolved in DMSO and injected into a reversed phase column (Phenomenex Sepra™ C18-E (Phenomenex, Inc., Torrance, CA, USA), 50 µm, 65 Å, 70 g). The column was eluted with a H_2_O: CH_3_CN linear gradient of decreasing polarity (15 mL/min; 20–60% acetonitrile in 42 min with a final isocratic step of 100% acetonitrile for 15 min), collecting 40 fractions of 15 mL. Fractions were evaporated to dryness in a centrifugal evaporator and then analyzed by LC/MS to locate the target 3-hydroxyquinaldic derivatives based on their UV and mass spectra.

3-Hydroxy-quinaldic acid (**4**, 77.0 mg) was identified as the unique component of fractions 10–11 by LC/MS and ^1^H NMR analysis.

Fractions 16–19 were dissolved in DMSO and purified by reversed-phase preparative HPLC (Agilent Zorbax SB-C8 PrepHT, 21.2 × 250 mm) eluting with a linear gradient of H_2_O-0.1% TFA: CH_3_CN-0.1% TFA (15 mL/min, 20–32% CH_3_CN in 40 min), to yield compound **1** (5.1 mg; *t*_R_: 24.0 min) and compound **2** (2.2 mg, *t*_R_: 20.4 min).

Fractions 28–31 were dissolved in DMSO and purified by reversed-phase preparative HPLC (Agilent Zorbax SB-C8 PrepHT, 21.2 × 250 mm) eluting with a linear gradient of H_2_O-0.1% TFA: CH_3_CN-0.1% TFA (15 mL/min, 20–50% CH_3_CN in 40 min) to yield compound **3** (1.7 mg; *t*_R_: 18.9 min) and the known compound **3**-hydroxyquinoline-2-carboxamide (**5**, 4.2 mg, *t*_R_: 20.9 min).

During the structural characterization of compound **3**, a spontaneous dimerization through a disulfide bond formation on standing on DMSO-*d*_6_ led to full conversion into compound **6**.

**Compound 1**: yellow solid; ${\left[\alpha \right]}_{D}^{20}$ + 42.9 (*c* 0.35, MeOH); UV (DAD) λ_max_ 232, 308, 360 nm; IR (ATR) ν_max_ 3341, 2946, 1660, 1531, 1187, 1138, 1018, 797 cm^−1^; for ^1^H and ^13^C NMR data see Table 1; (+)-ESI-TOFMS *m*/*z* 277.0821 [M + H]^+^ (calcd. for C_13_H_13_N_2_O_5_, 277.0824).

**Compound 2**: yellow solid; ${\left[\alpha \right]}_{D}^{20}$ − 9.6 (*c* 0.17, MeOH); UV (DAD) λ_max_ 232, sh 308, 360 nm; IR (ATR) ν_max_ 3343, 2946, 1658, 1532, 1185, 1135, 1019, 721 cm^−1^; for ^1^H and ^13^C NMR data see Table 1; (+)-ESI-TOFMS *m*/*z* 318.1088 [M + H]^+^ (calcd. for C_15_H_16_N_3_O_5_, 318.1090).

**Compound 3**: yellow solid; ${\left[\alpha \right]}_{D}^{20}$ + 3.3 (*c* 0.47, MeOH); UV (DAD) λ_max_ 230, 260, 308, 360 nm; IR (ATR) ν_max_ 3371, 2954, 1672, 1452, 1195, 1140, 1019, 845, 800, 724 cm^−1^; for ^1^H and ^13^C NMR data see Table 2; (+)-ESI-TOFMS *m*/*z* 360.0650 [M + H]^+^ (calcd. for C_16_H_14_N_3_O_5_S, 360.0654).

**Compound 6**: yellow solid; ${\left[\alpha \right]}_{D}^{20}$ − 8.4 (*c* 0.29, MeOH); UV (DAD) λ_max_ 230, 260, 308, 360 nm; IR (ATR) ν_max_ 3238, 2954, 2927, 1669, 1515, 1441, 1203, 1140, 840, 723 cm^−1^; for ^1^H and ^13^C NMR data see Table 2; (+)-ESI-TOFMS *m/z* 717.1063 [M + H]^+^ (calcd. for C_32_H_25_N_6_O_10_S_2_, 717.1073).

### 4.5. Hydrolysis and Marfey’s Analysis of Compounds ***1*** and ***2***

Samples (300 µg) of compounds **1** and **2** were separately dissolved in 0.6 mL of 6 N HCl and heated at 110 °C for 16 h in a sealed vial. The crude hydrolysates were evaporated to dryness under a nitrogen stream and each residue was dissolved in 100 µL of water. A 1% (w/v) solution (100 µL) of l-FDVA (Marfey’s reagent, *N*^α^-(2,4-dinitro-5-fluorophenyl)-l-valinamide) in acetone was added to an aliquot (50 µL) of a 50 mM solution of each amino acid standard (serine and aspartic acid, d, l, or dl mixture) and to the aqueous solution of each compound hydrolysate. After addition of 20 µL of 1 M NaHCO_3_ solution, each mixture was incubated for 60 min at 40 °C. The reactions were quenched by addition of 10 µL of 1 N HCl and the crude mixtures were diluted with 700 µL of acetonitrile and analyzed by ESI LC/MS on an Agilent 1100 single Quadrupole LC/MS. Separations were carried out on an Agilent Zorbax SB-C8 column (2.1 × 30 mm, 5 μm), maintained at 40 °C. A mixture of two solvents, A (10% CH_3_CN, 90% H_2_O) and B (90% CH_3_CN, 10% H_2_O), both containing 1.3 mM trifluoroacetic acid and 1.3 mM ammonium formate, was used as the mobile phase under a linear gradient (10–50% B in 6 min) at a flow rate of 0.3 mL/min. Retention times (min) for the derivatized (L-FDVA) amino acid standards under these chromatographic conditions were as follows: L-Ser: 3.02; D-Ser: 3.42; L-Glu: 3.33 D-Glu: 3.83. Retention times (min) for the observed peaks in the HPLC trace of the l-FDVA-derivatized hydrolysis products of compounds **1** and **2**, in agreement with the presence of L-Ser (3.01) in **1** and L-Glu (3.37) in **2**.

### 4.6. Oxidation of Compound ***3*** with Performic Acid and Marfey’s Analysis

Compound **3** (0.2 mg) was treated with H_2_O_2_-HCOOH (1:4) (0.8 mL) at 0 °C and the reaction mixture was allowed to reach room temperature over 2 h. The oxidation of **3** to the corresponding cysteic acid derivative was confirmed by LC-HRMS (Figures S35 and S36). Solvents were evaporated to dryness under a N_2_ stream and the residue was hydrolyzed with 0.6 mL of HCl 6 N at 110 °C for 18 h. The hydrolyzed product and the standard amino acid l-cysteic were separately derivatized with l-FDVA or d-FDVA under an analogous procedure to that used for Marfey’s analysis of compounds **1** and **2**. Separations were carried out on a Water Atlantis T3 C18 column (4.6 × 100 mm, 5 μm) maintained at 40 °C. A mixture of water (A) and acetonitrile (B), both containing 0.1% HCOOH, was used as the mobile phase under a linear gradient (25–30% B in 14 min; then, 100% B) at a flow rate of 1.0 mL/min. Retention times (min) for the derivatized (l-FDVA or d-FDVA) amino acid standard l-cysteic acid under these chromatographic conditions were as follows: l-cysteic: 9.38; d-cysteic (enantiomer of l-cysteic-d-FDVA): 8.51. Retention time (9.56 min) for the peak observed in the HPLC trace of the l-FDVA-derivatized hydrolysis of the oxidation product of compound **3** was in agreement with the presence of l-cysteic acid, and therefore with the presence of l-cysteine in compounds **3** and **6**.

### 4.7. Antibacterial Activity Assays

The antibacterial activities of the compounds were evaluated using sequential 2-fold serial dilutions of each compound in DMSO to provide 10 concentrations starting at 64 µg/mL for all of the assays. Activity was measured against one Gram-positive (methicillin-resistant *S. aureus* MB5393) and two Gram-negative (*E. coli* MB2884 and *A. baumannii* MB5973) bacterial strains, as previously reported [32].

### 4.8. Cytotoxicity Assays

Cytotoxic activity against the HepG2 human tumor cell line was tested as previously described [33], using sequential 2-fold serial dilutions of each compound in DMSO to provide 10 concentrations starting at 20 µg/mL.

## Figures and Tables

**Table 1:** NMR spectroscopic data (DMSO-*d*_6_, 500 MHz) for compounds **1** and **2**.

Position	1		2	
	δ_C_, Type	δ_H_ (*J* in Hz)	δ_C_, Type *	δ_H_ (*J* in Hz)
1	167.8, C		168.7, C	
2	134.6, C		135.4, C	
3	153.1, C		153.4, C	
4	120.3, CH	7.89, s	120.4, CH	7.87, s
5	131.6, CH		131.7, CH	
6	126.7, CH	7.91, dd (7.6, 1.8)	127.0, CH	7.91, d (7.6, 1.7)
7	128.9, CH 127.8, CH	7.63, m	129.2, CH 128.0, CH	7.65, m
8		7.66, m		7.67, m
9	129.0, CH	8.05, d (8.3)	129.4, CH	8.05, d (8.0)
10	140.8, C		141.2, C	
3-OH		12.01, s		12.05, s
1′	171.1, C		172.7, C	
2′	54.6, CH	4.61, ddd (8.1, 4.2, 3.3)	52.3, CH	4.53, ddd (8.6, 8.0, 3.7)
3′	60.8, CH_2_	3.98, dd (11.2, 4.2) 3.89, dd (11.2, 3.3)	26.5, CH_2_	2.22, m 2.11, m
4′	-	-	31.6, CH_2_	2.22, m, 2H
5′			173.8, C	
NH		9.19, d (8.1)		9.53, d (8.8)
COOH		13.11, br s		-
NH_2_		-		7.32, br s/6.80, br s

* Chemical shifts determined using HSQC and HMBC experiments.

**Table 2:** NMR spectroscopic data (DMSO-*d_6_*, 500 MHz) for compounds **3** and **6**.

Position	3		6	
	δ_C_, Type *	δ_H_ (*J* in Hz)	δ_C_, Type *	δ_H_ (*J* in Hz)
1	159.1, C		159.1, C	
2	134.5, C		134.1, C	
3	150.2, C		150.2, C	
4	119.8, CH	7.96, br s	119.7, CH	7.92, br s
5	130.4, C		130.4, C	
6	127.0, CH	7.89, dd (7.4, 1.8)	127.0, CH	7.89, dd (7.1, 1.6)
7	128.9, CH 128.0, CH	7.59, m	128.9, CH 128.0, CH	7.59, ddd (7.1, 6.8, 1.4)
8		7.63, m		7.62, ddd (8.1, 6.8, 1.6)
9	129.4, CH	8.03, br d (7.8)	129.4, CH	8.00, br d (8.1)
10	142.5, C		142.4, C	
3-OH		10.61, s		10.45, s
1′	159.2, C		159.2, C	
2′	136.2, C		136.0, C	
3′	143.6, CH	8.96, s	143.8, CH	8.90, s
1″	172.0, C		172.0, C	
2″	55.3, CH	4.58, m	51.6, CH	4.79, ddd (8.5, 4.5, 4.3)
3″	25.6, CH_2_	3.08, m 2.96, m	39.3, CH_2_	3.38, m 3.19, dd (12.6, 4.5)
NH				9.20, d (8.5)
COOH				13.09, br s

* Chemical shifts determined using HSQC and HMBC experiments.

## Supplementary Materials

The following are available online at http://www.mdpi.com/1660-3397/16/10/371/s1, Figure S1: UV spectrum of compound **1**; Figure S2: ESI-TOF spectrum of compound **1**; Figure S3: ^1^H NMR spectrum (DMSO-*d*_6_, 500 MHz) of compound **1**; Figure S4: ^13^C NMR spectrum (DMSO-*d*_6_, 125 MHz) of compound **1**; Figure S5: COSY spectrum of compound **1**; Figure S6: HSQC spectrum of compound **1**; Figure S7: HMBC spectrum of compound **1**; Figure S8: UV spectrum of compound **2**; Figure S9: ESI-TOF spectrum of compound **2**; Figure S10: ^1^H NMR spectrum (DMSO-*d*_6_, 500 MHz) of compound **2**; Figure S11: COSY spectrum of compound **2**; Figure S12: HSQC spectrum of compound **2**; Figure S13: HMBC spectrum of compound **2**; Figure S14: UV spectrum of compound **3**; Figure S15: ESI-TOF spectrum of compound **3**; Figure S16: ^1^H NMR spectrum (DMSO-*d*_6_, 500 MHz) of compound **3**; Figure S17: COSY spectrum of compound **3**; Figure S18: HSQC spectrum of compound **3**; Figure S19: HMBC spectrum of compound **3**; Figure S20: UV spectrum of compound **4**; Figure S21: ESI-TOF spectrum of known compound **4**; Figure S22: ^1^H NMR spectrum (CDCl_3_, 500 MHz) of known compound **4**; Figure S23: UV spectrum of compound **5**; Figure S24: ESI-TOF spectrum of known compound **5**; Figure S25: ^1^H NMR spectrum (DMSO-*d*_6_, 500 MHz) of known compound **5**; Figure S26: UV spectrum of compound **6**; Figure S27: ESI-TOF spectrum of compound **6**; Figure S28: ^1^H NMR spectrum (DMSO-*d*_6_, 500 MHz) of compound **6**; Figure S29: COSY spectrum of compound **6**; Figure S30: HSQC spectrum of compound **6**; Figure S31: HMBC spectrum of compound **6**; Figure S32: ^1^H-NMR (DMSO-*d*_6_, 500 MHz) time-course conversion of **3** into **6**; Figure S33: HPLC traces of Marfey’s analysis of compound **1**; Figure S34: HPLC traces of Marfey’s analysis of compound **2**; Figure S35: LC-HRMS analysis of the oxidation crude of compound **3**; Figure S36: HRMS-MS spectrum of the oxidation product of compound **3**; Figure S37: HPLC traces of l- and d-FDVA derivatives of standard L-cysteic acid; Figure S38: HPLC traces of Marfey’s analysis of the oxidation product of compound **3**.
