# Supplementary material for: New 3-Hydroxyquinaldic Acid Derivatives from Cultures of the Marine Derived Actinomycete Streptomyces cyaneofuscatus M-157
Source: Mar Drugs. 2018 Oct 8;16(10):371. doi: 10.3390/md16100371 (PMC6212950; doi:10.3390/md16100371)
Supplement: Supplementary file 1 [file marinedrugs-16-00371-s001.pdf]

New 3-Hydroxyquinaldic Acid Derivatives from Cultures of the Marine Derived Actinomycete *Streptomyces cyaneofuscatus* M-157

Francisco Javier Ortiz-López [1,†], Elsa Alcalde [1,†], Aida Sarmiento-Vizcaíno [2], Caridad Díaz [1], Bastien Cautain [1], Luis A. García [3], Gloria Blanco [2,*] and Fernando Reyes [1,*]

[1] Fundación MEDINA, Centro de Excelencia en Investigación de Medicamentos Innovadores en Andalucía, Avda. del Conocimiento 3, Parque Tecnológico de Ciencias de la Salud, E-18016 Granada, Spain; javier.ortiz@medinaandalucia.es (F.J.O.-L.); elsaalcalde@uma.es (E.A.); caridad.diaz@medinaandalucia.es (C.D.); bastien.cautain@medinaandalucia.es (B.C.)

[2] Departamento de Biología Funcional, Área de Microbiología, and Instituto Universitario de Oncología del Principado de Asturias, Universidad de Oviedo, 33006 Oviedo, Spain; UO209983@uniovi.es (A.S.-V.)

[3] Departamento de Ingeniería Química y Tecnología del Medio Ambiente, Área de Ingeniería Química, Universidad de Oviedo, 33006 Oviedo, Spain; luisag@uniovi.es (L.A.G.)

* Correspondence: gbb@uniovi.es (G.B.); fernando.reyes@medinaandalucia.es (F.R.); Tel.: +34-985-103-205 (G.B.); +34-958-993-965 (F.R.)

† These authors contributed equally to this work.

List of supplementary materials

Figure S1. UV spectrum of compound **1**.

Figure S2. ESI-TOF spectrum of compound **1**.

Figure S3. ^{1}H NMR spectrum (DMSO-d_6, 500 MHz) of compound **1**.

Figure S4. ^{13}C NMR spectrum (DMSO-d_6, 125 MHz) of compound **1**.

Figure S5. COSY spectrum of compound **1**.

Figure S6. HSQC spectrum of compound **1**.

Figure S7. HMBC spectrum of compound **1**.

Figure S8. UV spectrum of compound **2**.

Figure S9. ESI-TOF spectrum of compound **2**.

Figure S10. ^{1}H NMR spectrum (DMSO-d_6, 500 MHz) of compound **2**.

Figure S11. COSY spectrum of compound **2**.

Figure S12. HSQC spectrum of compound **2**.

Figure S13. HMBC spectrum of compound **2**.

Figure S14. UV spectrum of compound **3**.

Figure S15. ESI-TOF spectrum of compound **3**.

Figure S16. ^{1}H NMR spectrum (DMSO-d_6, 500 MHz) of compound **3**.

Figure S17. COSY spectrum of compound **3**.

Figure S18. HSQC spectrum of compound **3**.

Figure S19. HMBC spectrum of compound **3**.

Figure S20. UV spectrum of compound **4**.

Figure S21. ESI-TOF spectrum of known compound **4**.

Figure S22. ^{1}H NMR spectrum ($CDCl_3$, 500 MHz) of known compound **4**.

Figure S23. UV spectrum of compound **5.**

Figure S24. ESI-TOF spectrum of known compound **5.**

Figure S25. ^{1}H NMR spectrum (DMSO-d_6, 500 MHz) of known compound **5.**

Figure S26. UV spectrum of compound **6**.

Figure S27. ESI-TOF spectrum of compound **6**.

Figure S28. ^{1}H NMR spectrum (DMSO-d_6, 500 MHz) of compound **6**.

Figure S29. COSY spectrum of compound **6**.

Figure S30. HSQC spectrum of compound **6**.

Figure S31. HMBC spectrum of compound **6**.

Figure S32. ^{1}H-NMR (DMSO-*d6*, 500 MHz) time-course conversion of **3** into **6**.

Figure S33: HPLC traces of Marfey´s analysis of compound **1**.

Figure S34: HPLC traces of Marfey´s analysis of compound **2**.

Figure S35: LC-HRMS analysis of the oxidation crude of compound **3.**

Figure S36: HRMS-MS spectrum of the oxidation product of compound **3**.

Figure S37: HPLC traces of L- and D-FDVA derivatives of standard L-cysteic acid**.**

Figure S38: HPLC traces of Marfey´s analysis of the oxidation product of compound **3**.

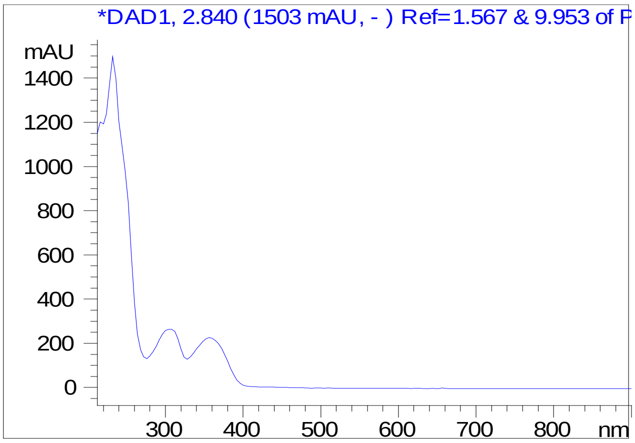

Figure S1. UV spectrum of compound **1**.

ISCID=75 eV

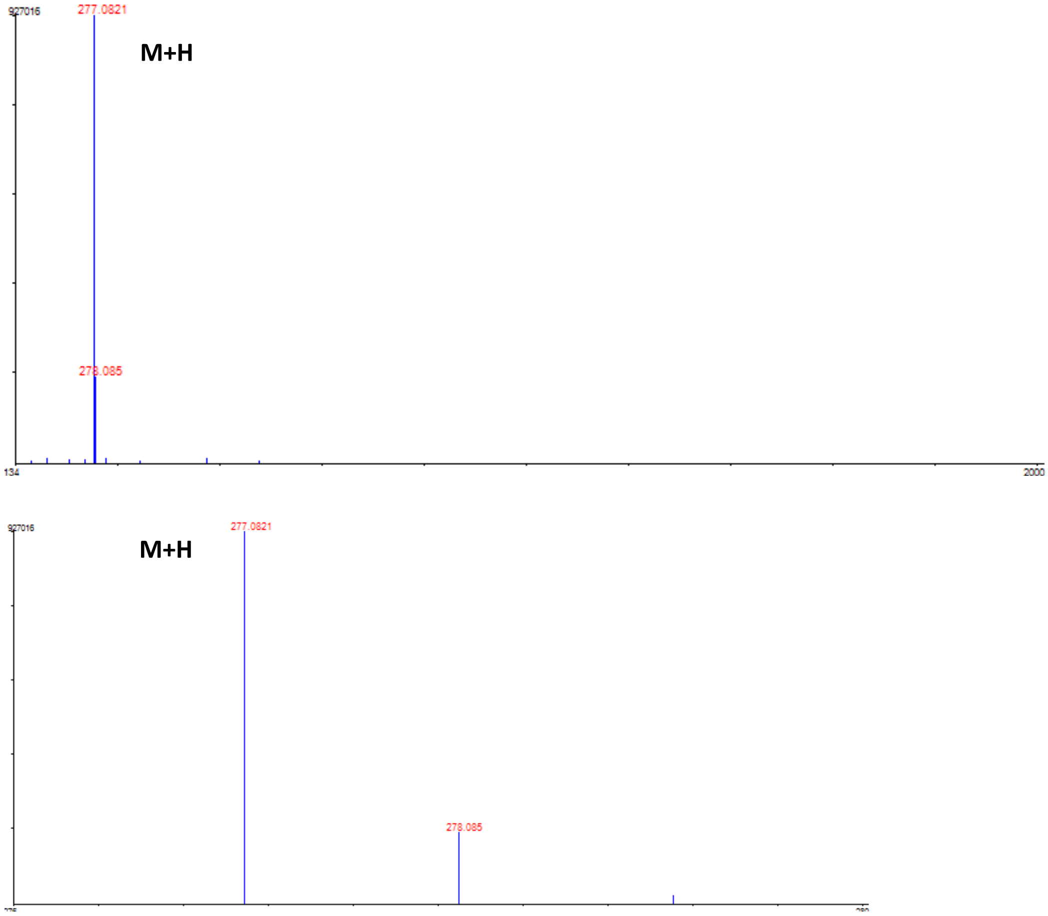

Figure S2. ESI TOF spectrum of compound **1**.

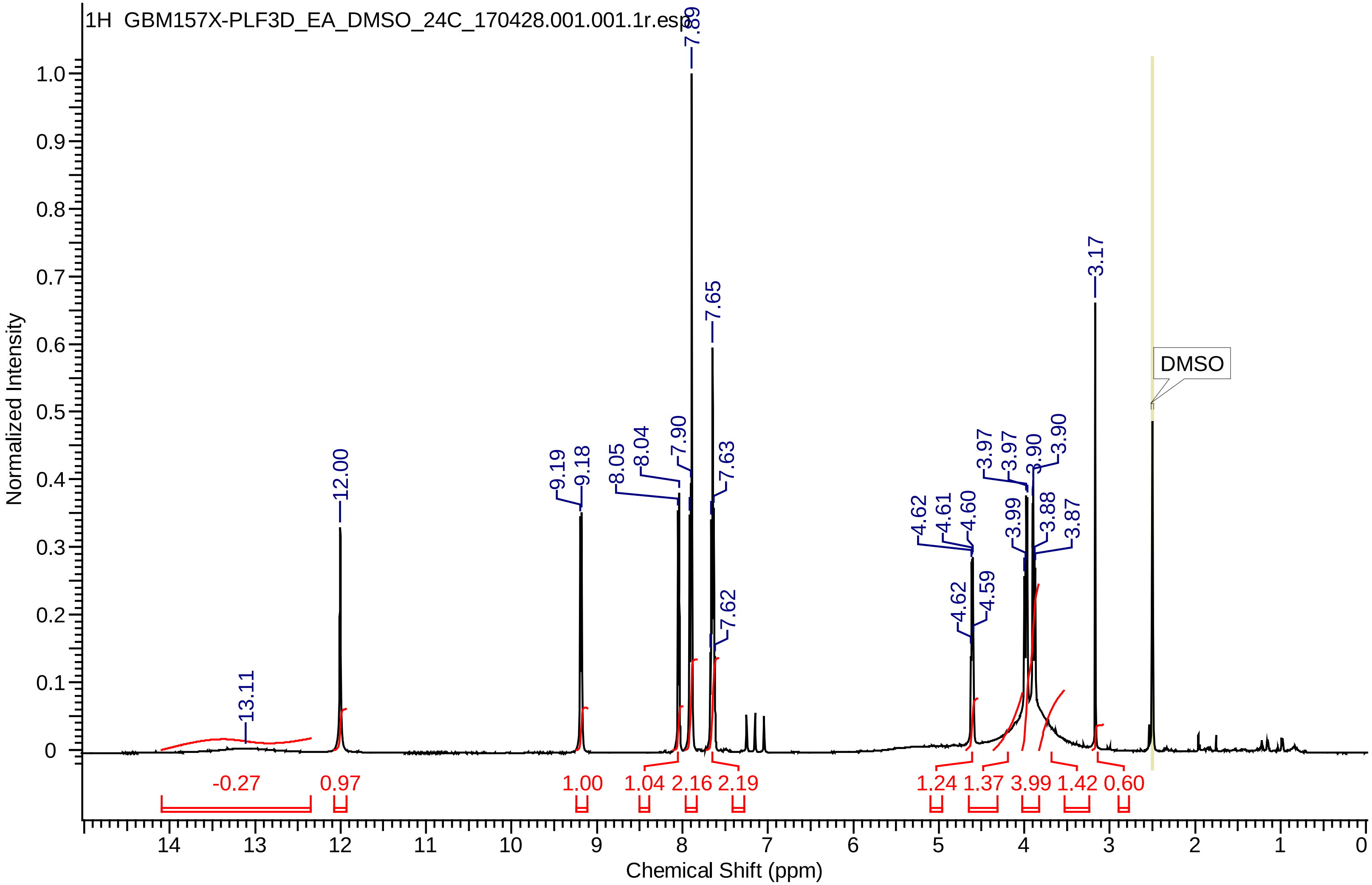

Figure S3. ^{1}H NMR (DMSO-*d6*, 500 MHz) of compound **1**.

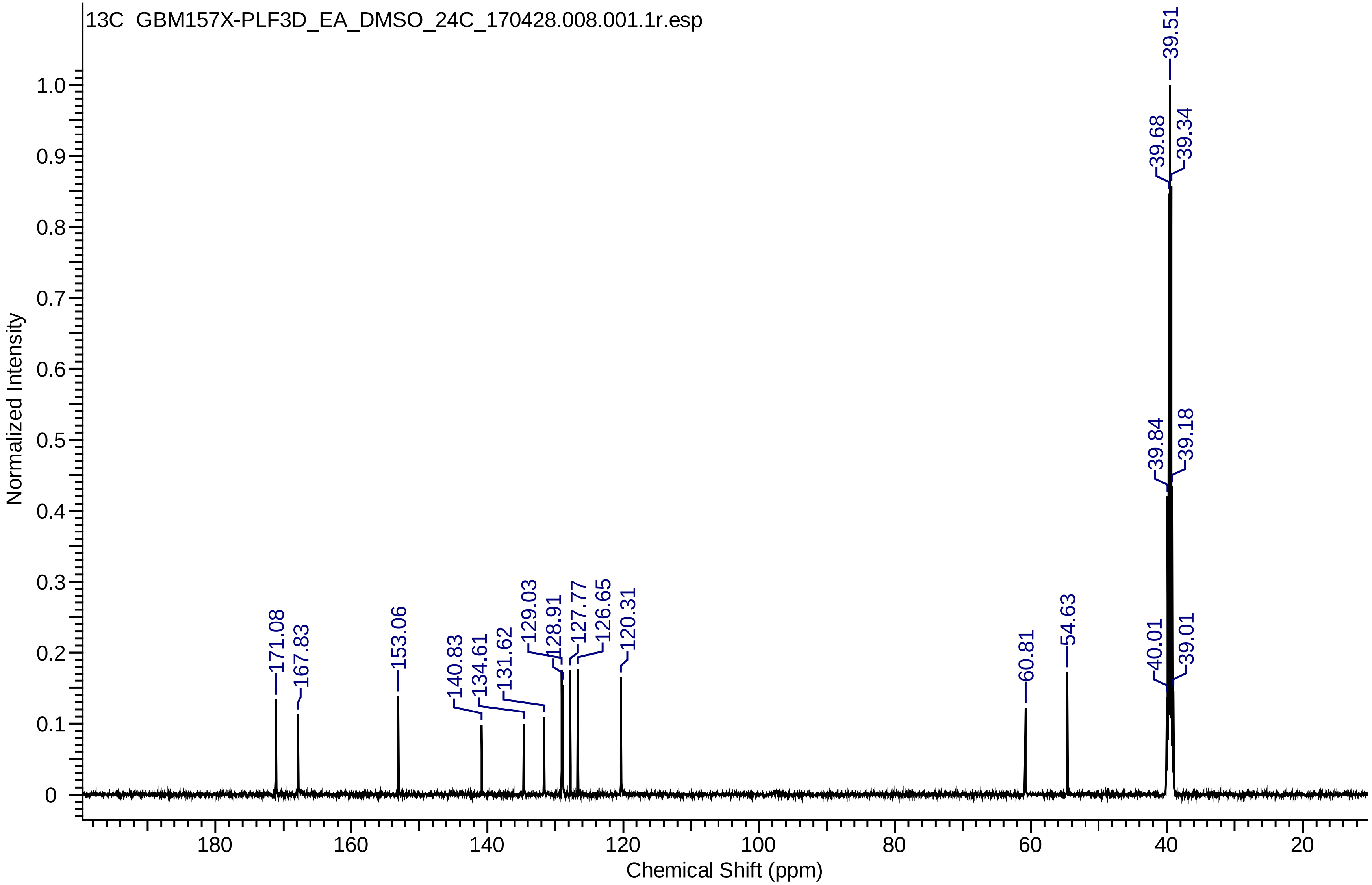

Figure S4. ^{13}C NMR (DMSO-*d6*, 125 MHz) of compound **1**.

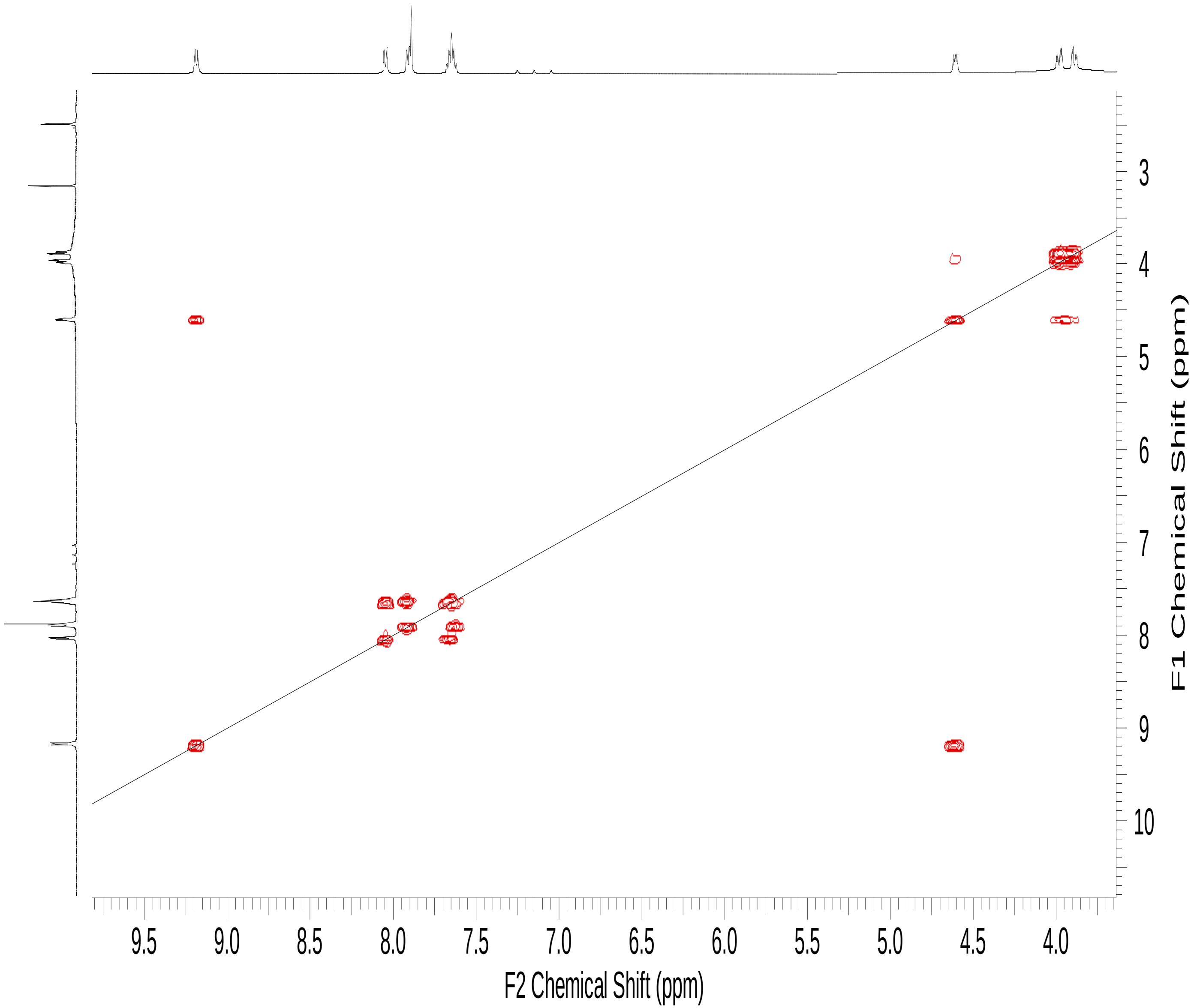

Figure S5. COSY spectrum of compound **1**.

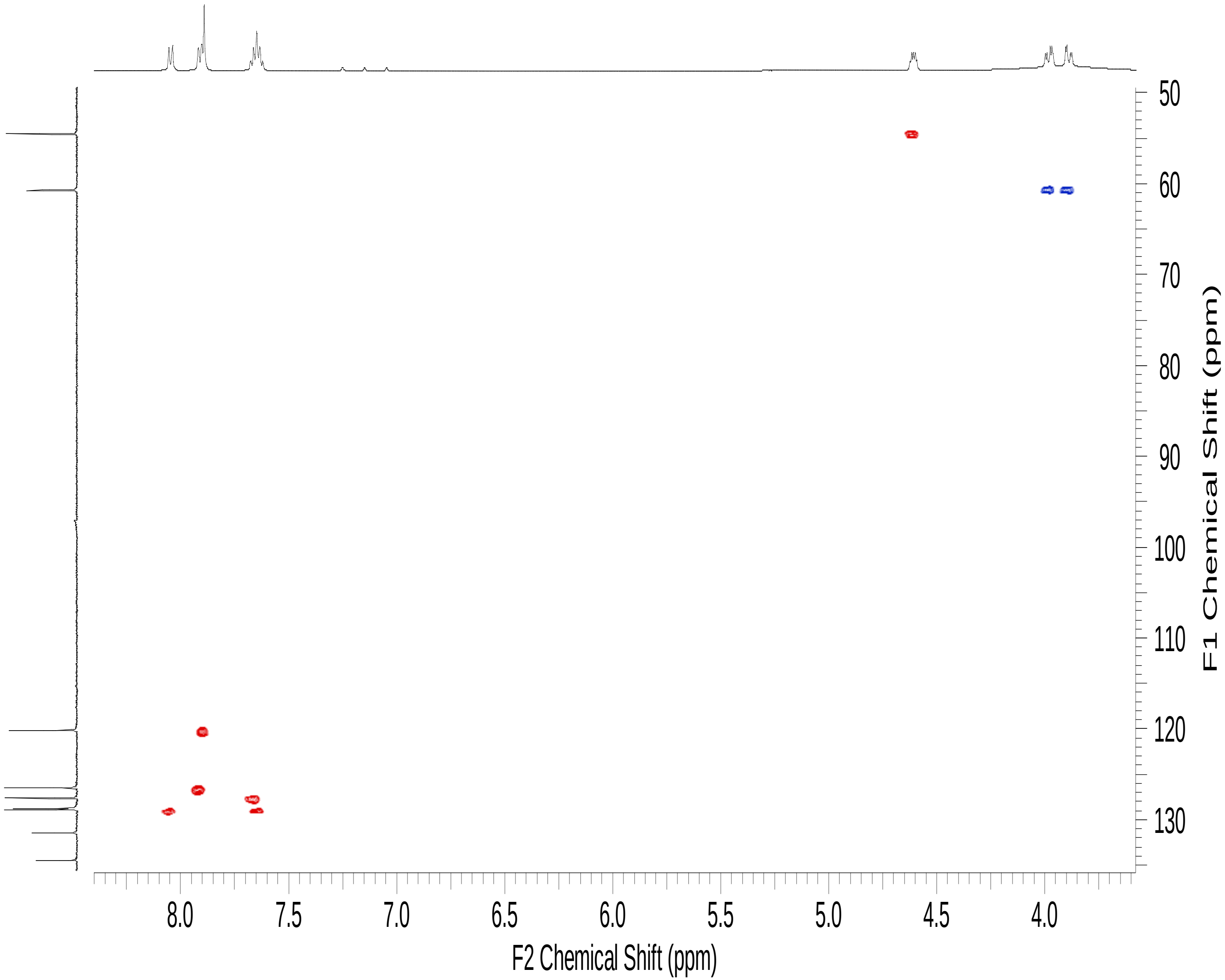

Figure S6. HSQC spectrum of compound **1**.

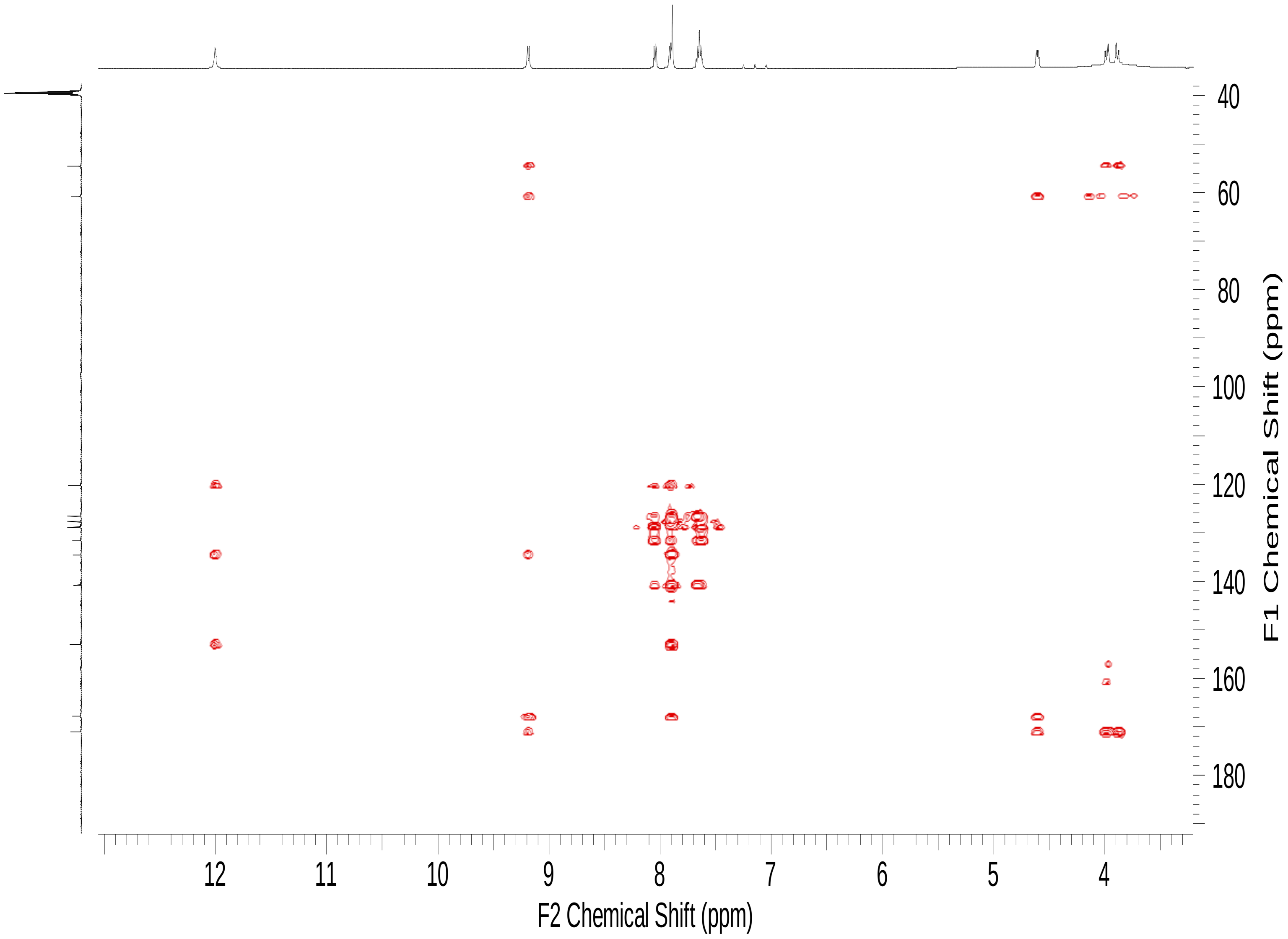

Figure S7. HMBC spectrum of compound **1**.

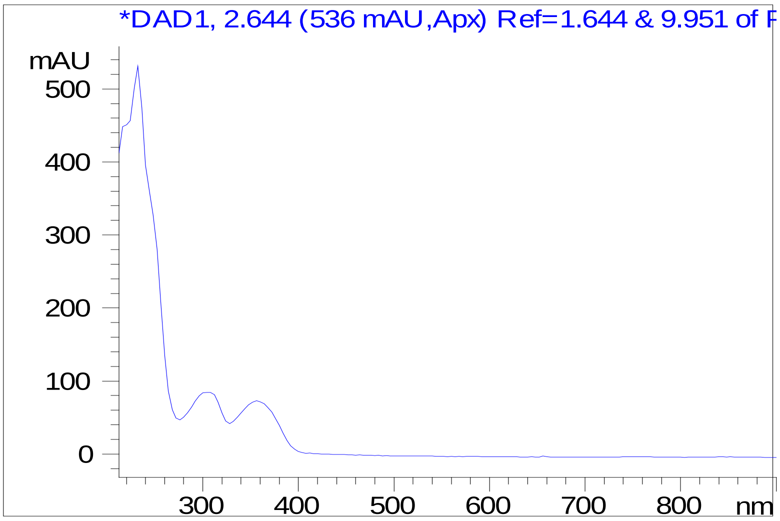

Figure S8. UV spectrum of compound **2**.

ISCID=75 eV

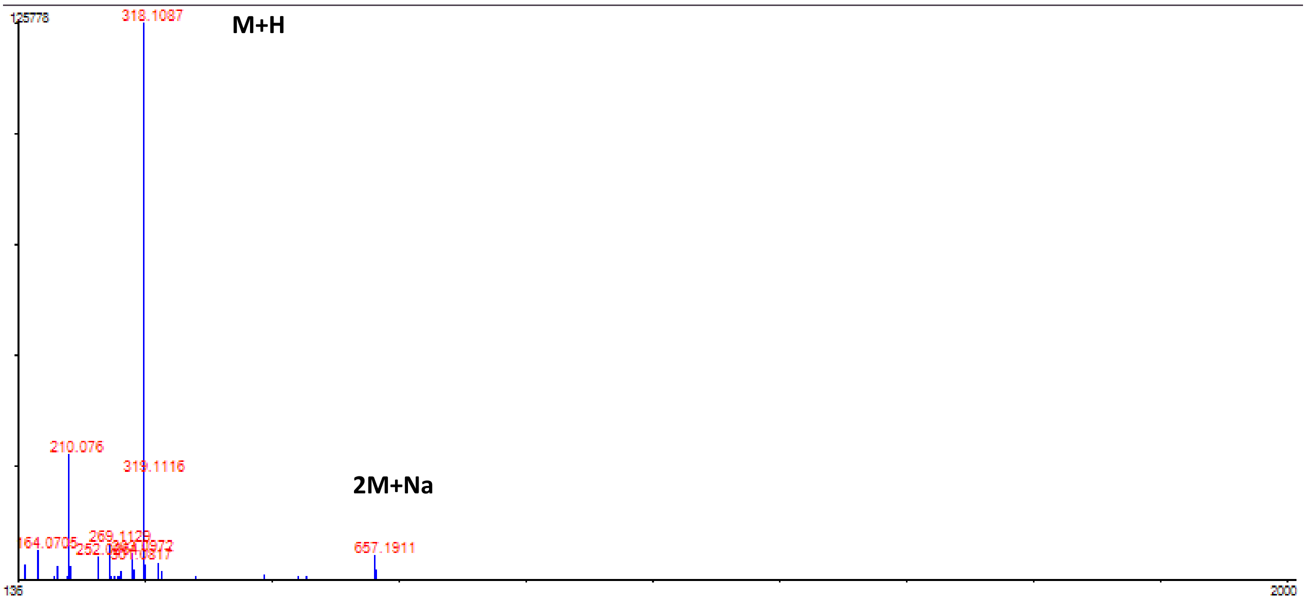

Figure S9. ESI TOF spectrum of compound **2**.

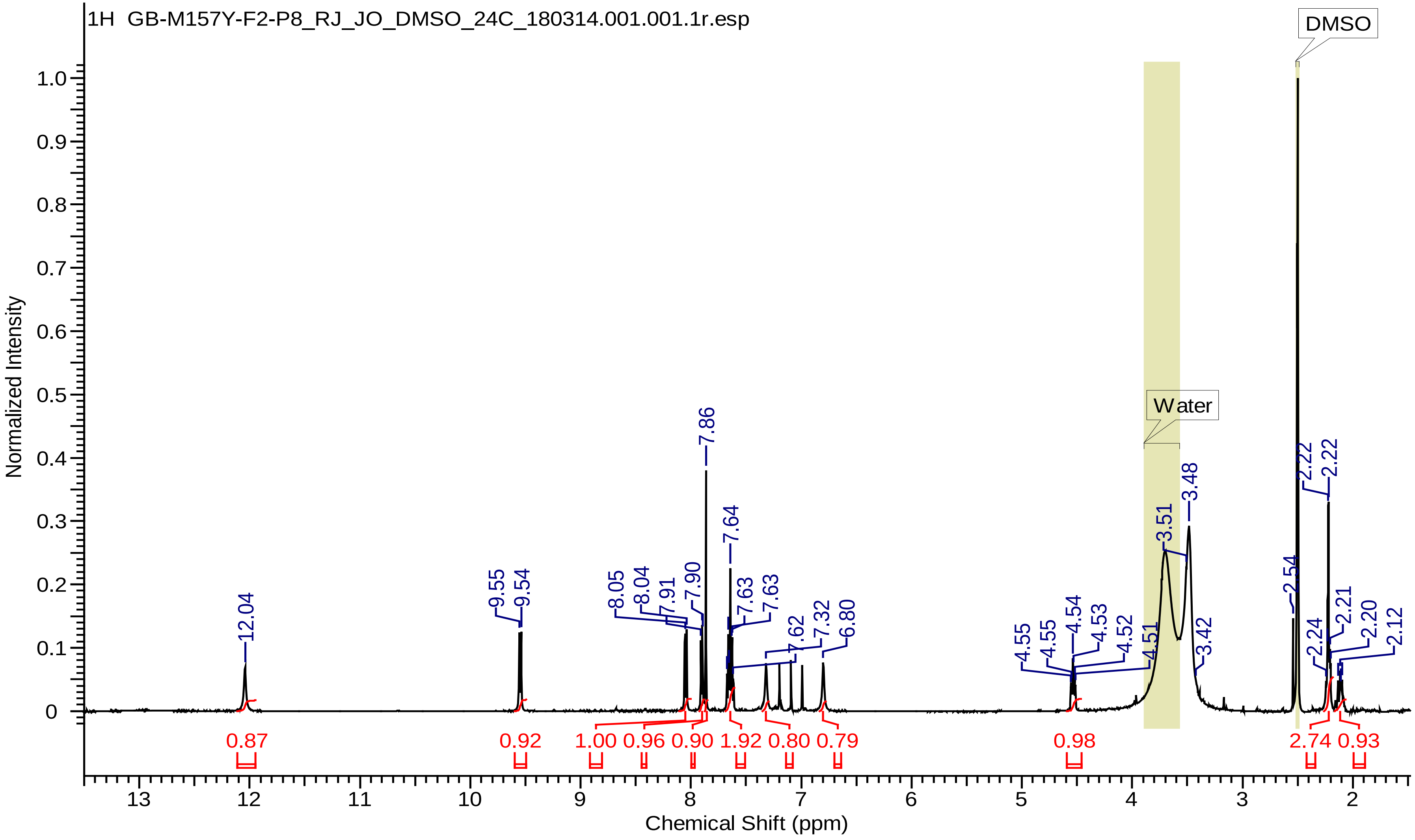

Figure S10. ^{1}H NMR (DMSO-*d6*, 500 MHz) of compound **2**.

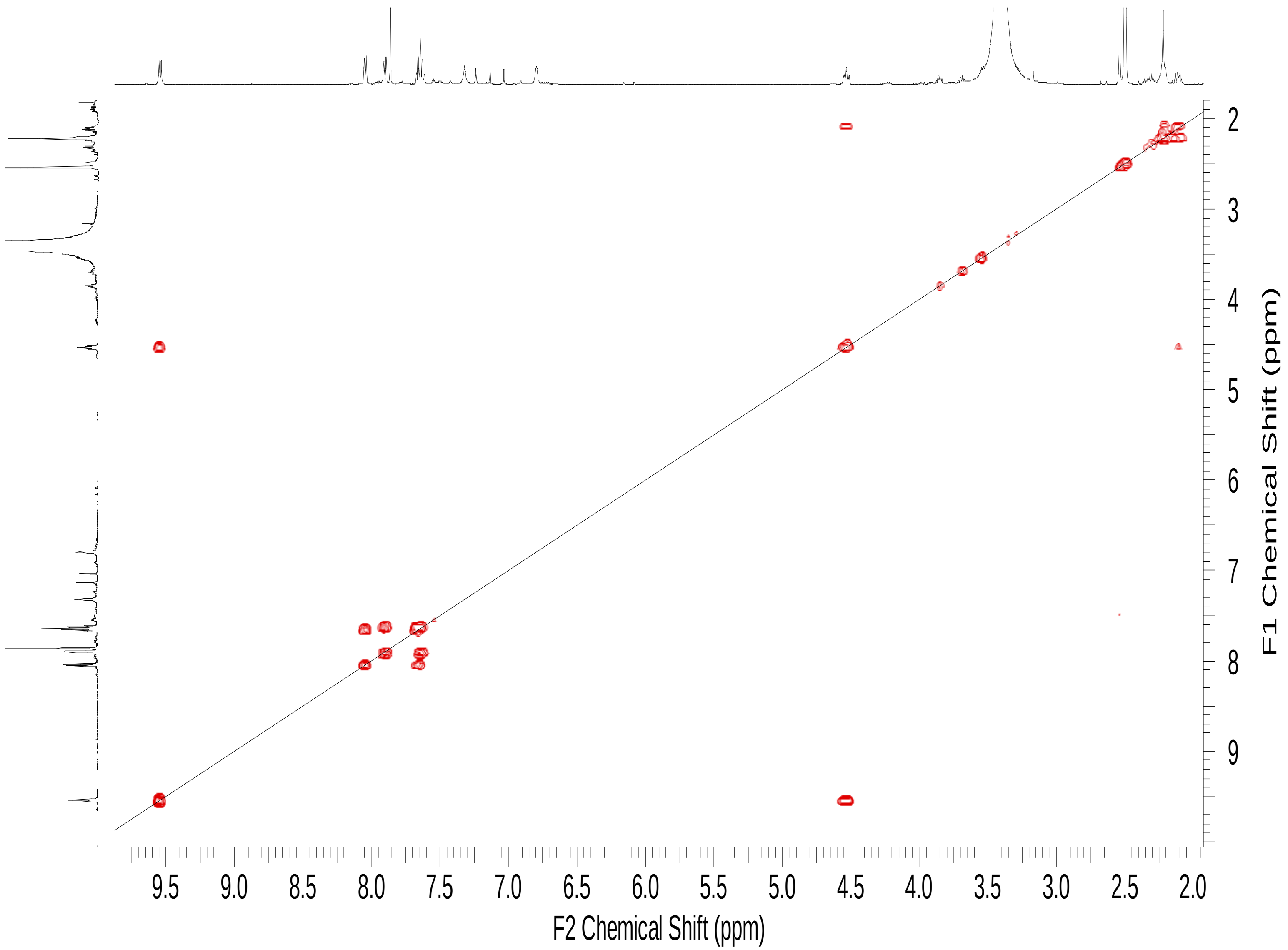

Figure S11. COSY spectrum of compound **2**.

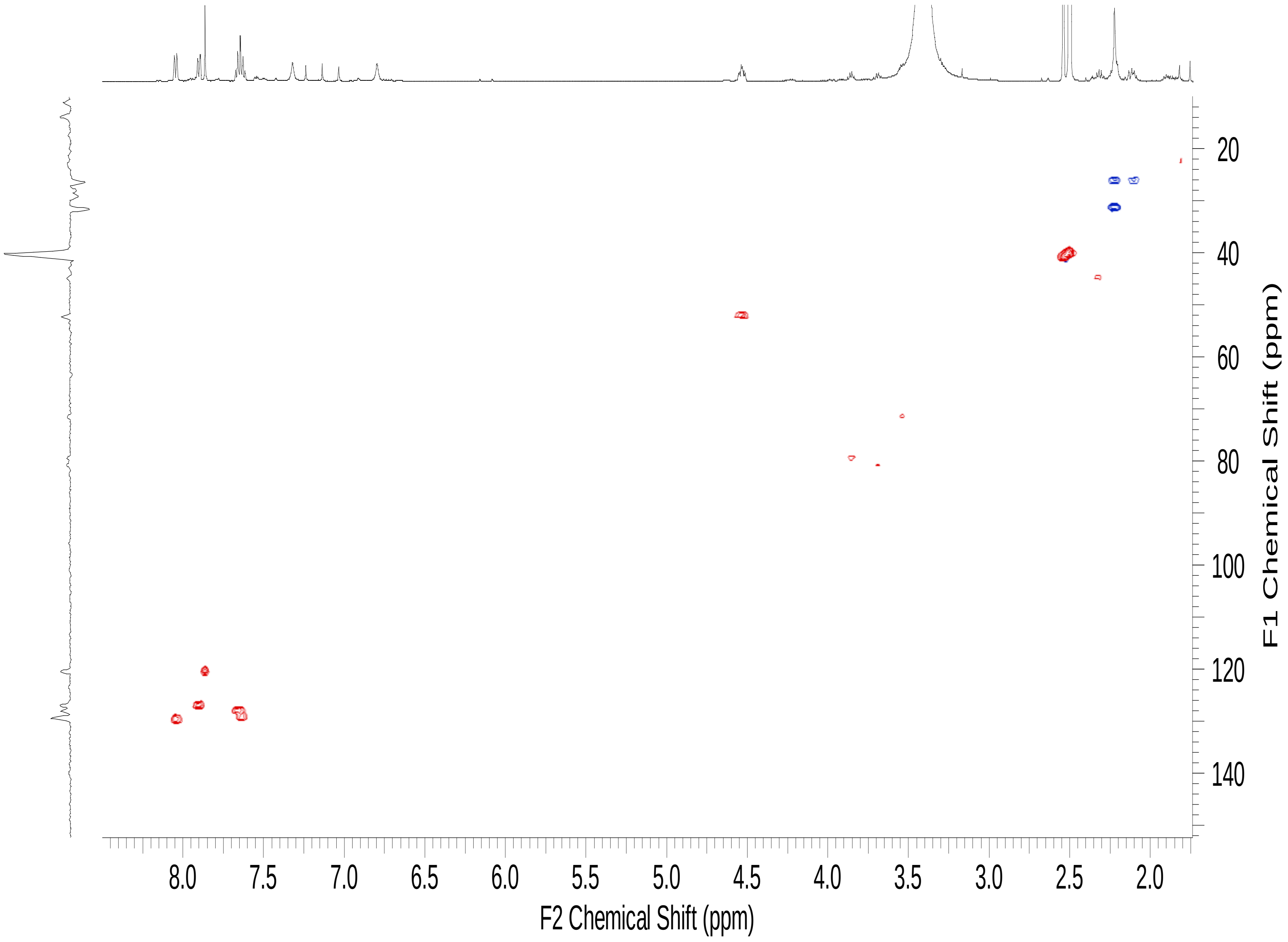

Figure S12. HSQC spectrum of compound **2**.

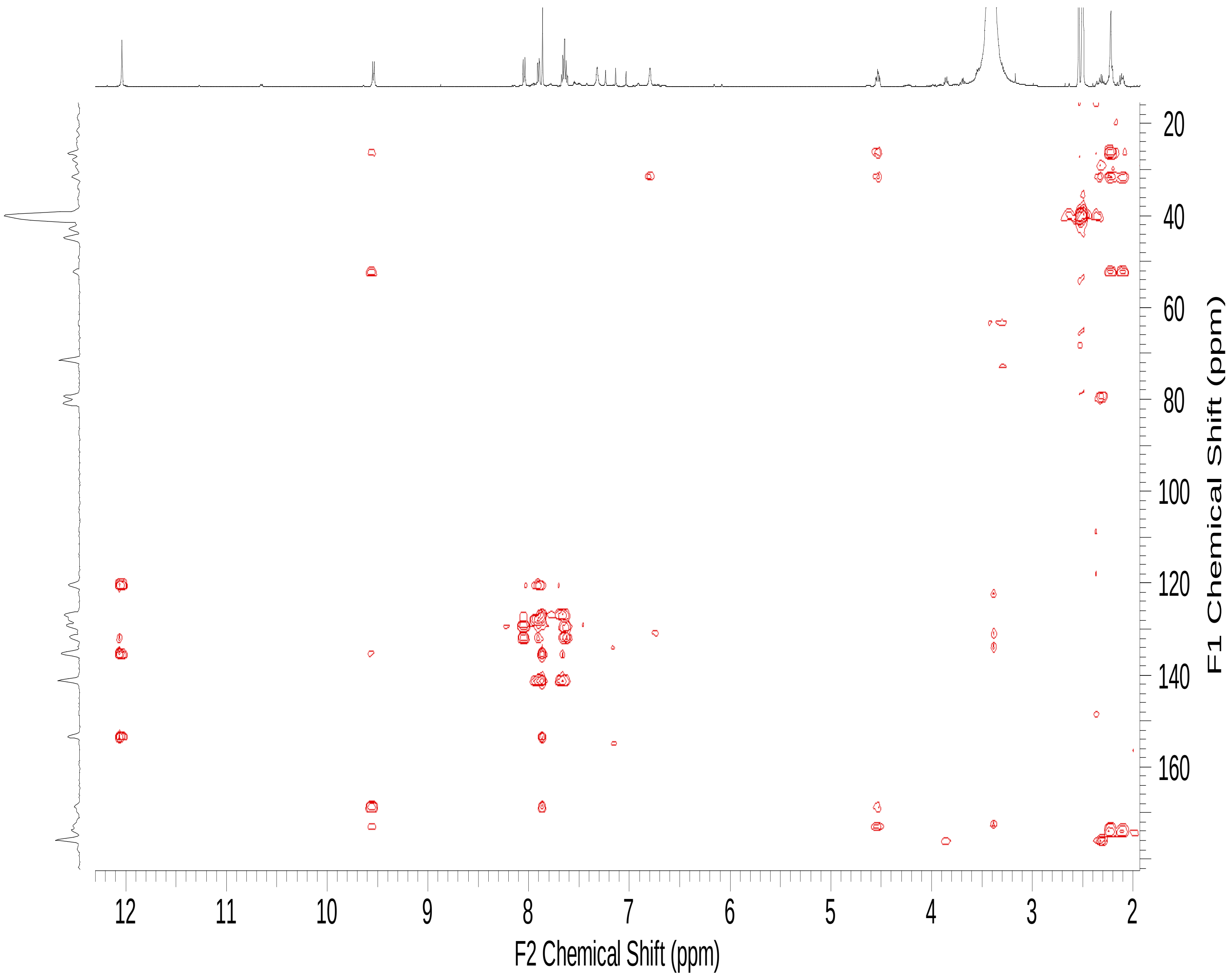

Figure S13. HMBC spectrum of compound **2**.

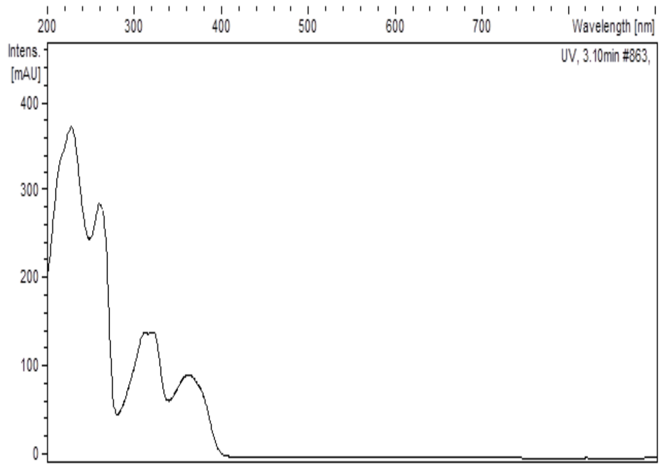

Figure S14. UV spectrum of compound **3**.

ISCID=75 eV

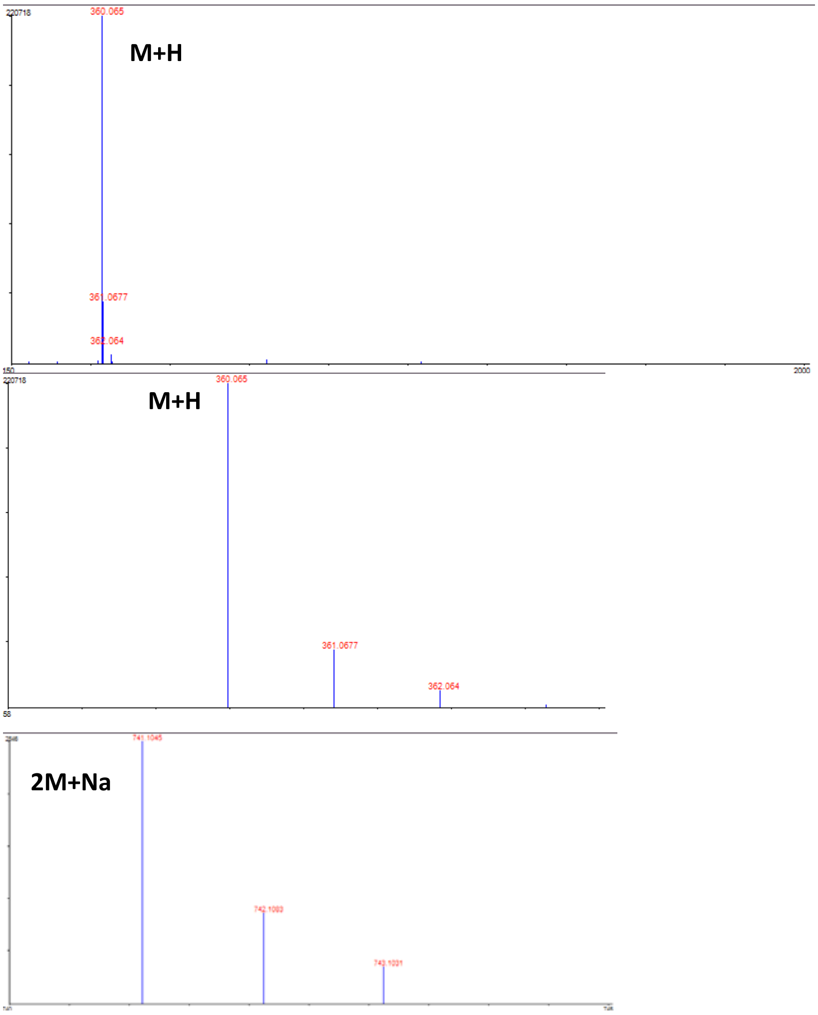

Figure S15. ESI TOF spectrum of compound **3**.

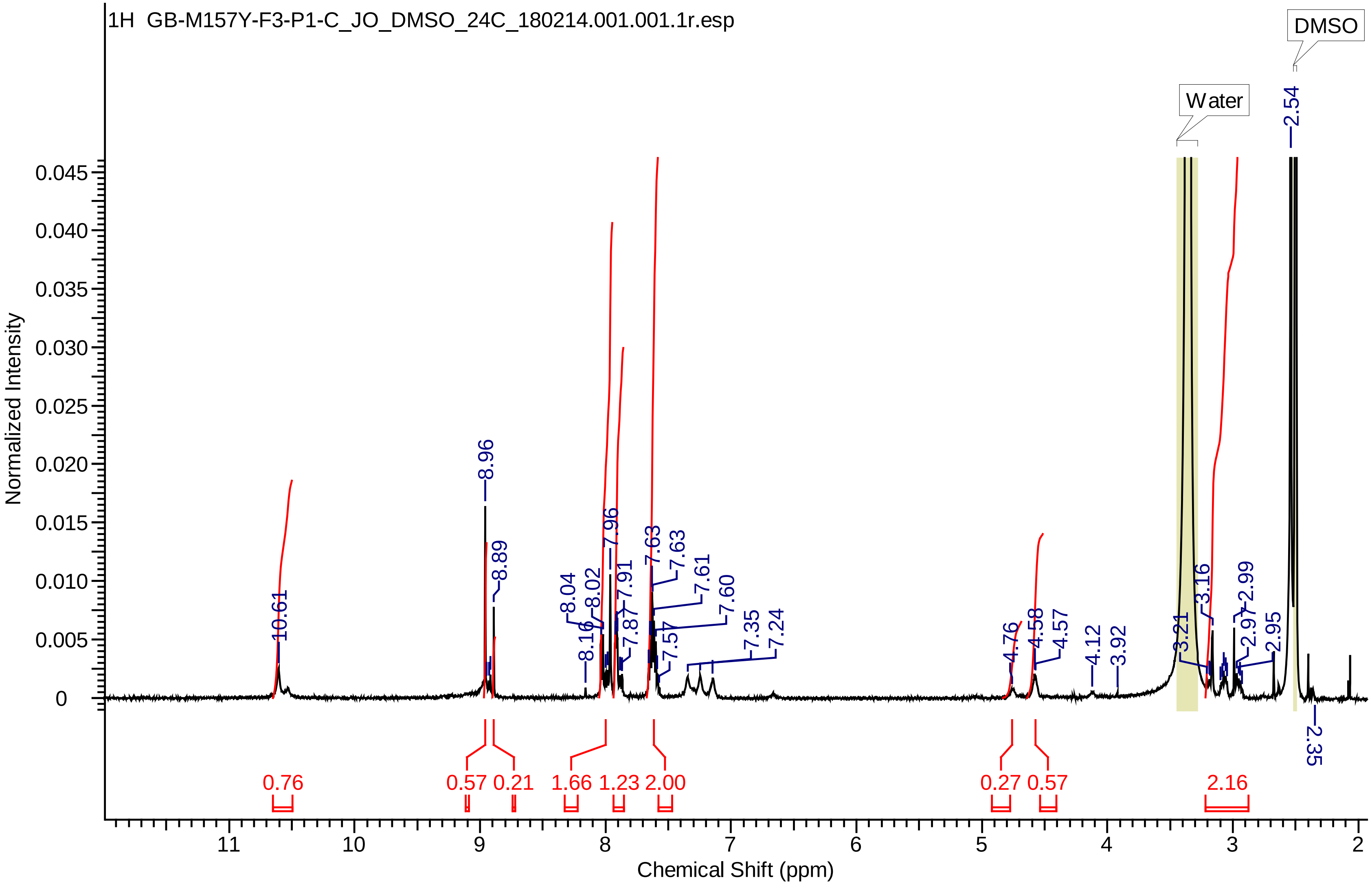

Figure S16. ^{1}H NMR (DMSO-*d6*, 500 MHz) of compound **3**.

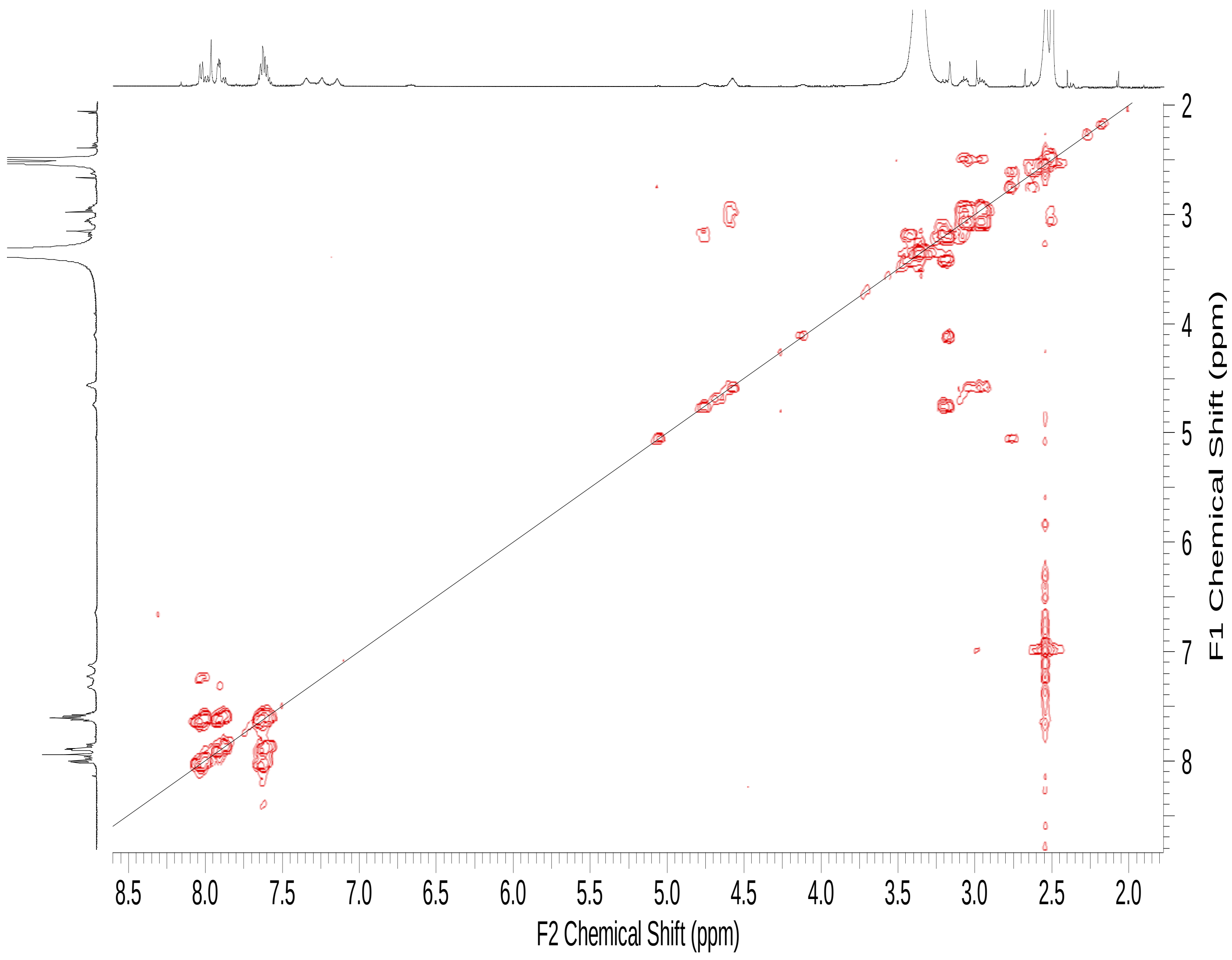

Figure S17. COSY spectrum of compound **3**.

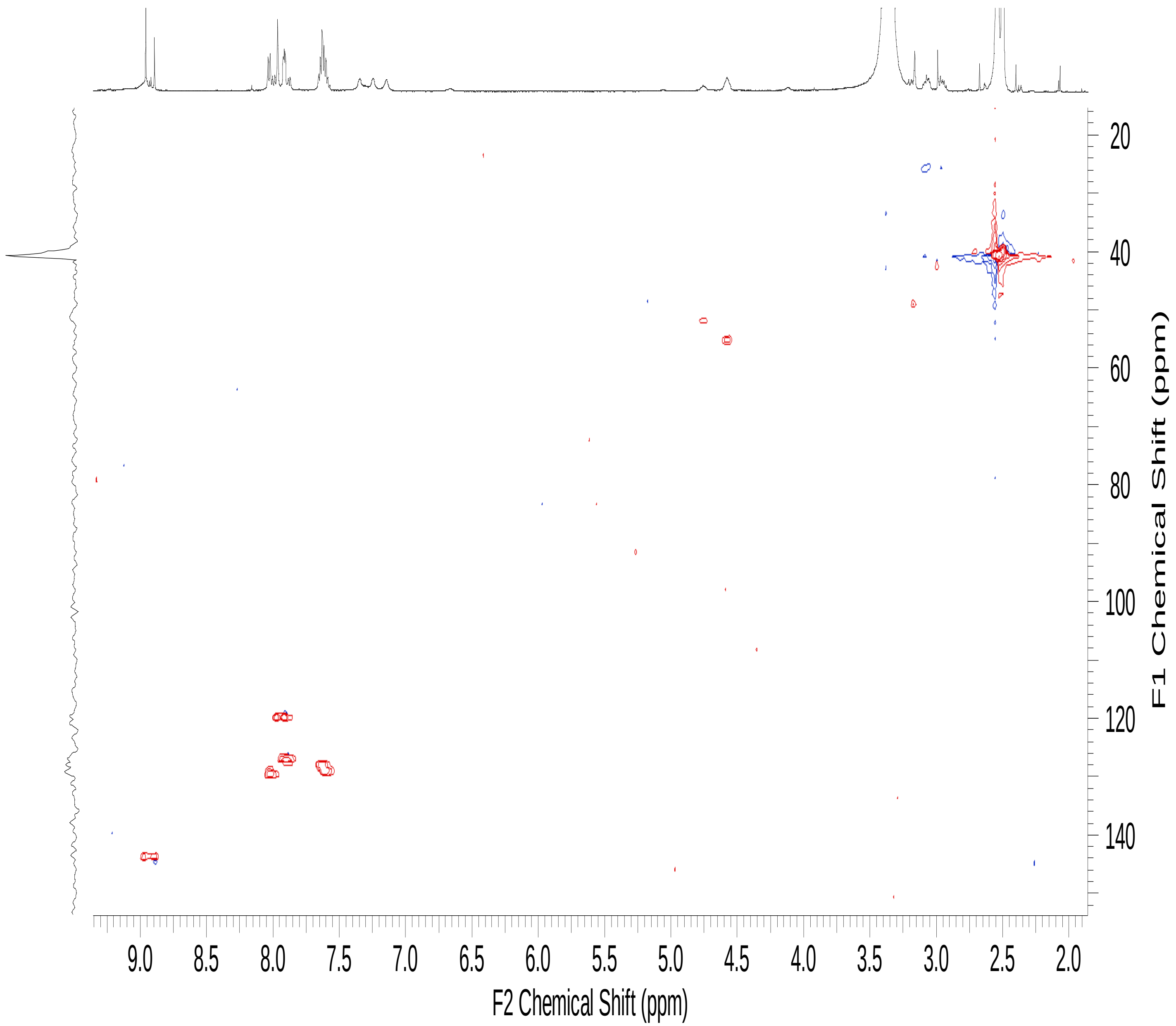

Figure S18. HSQC spectrum of compound **3**.

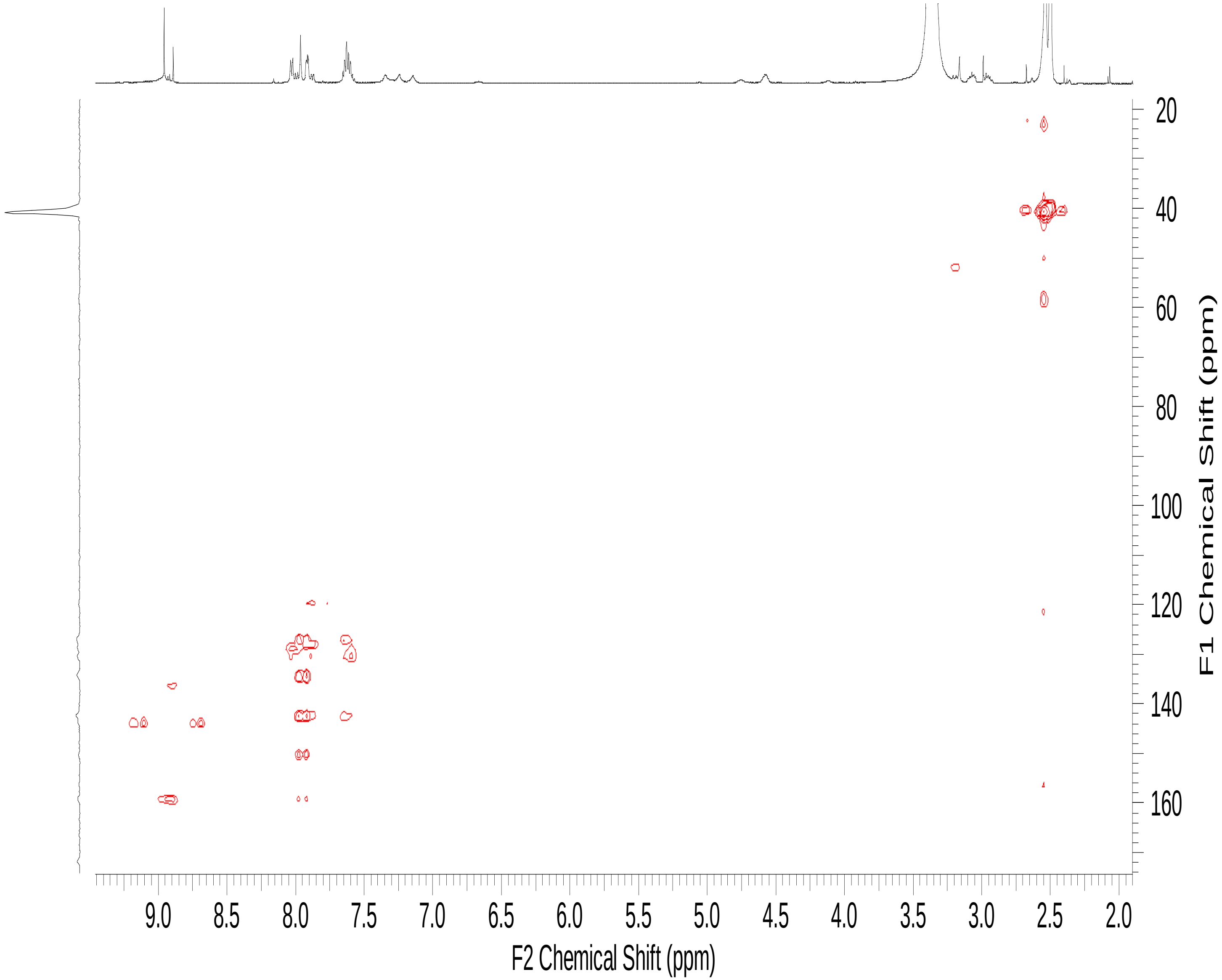

Figure S19. HMBC spectrum of compound **3**.

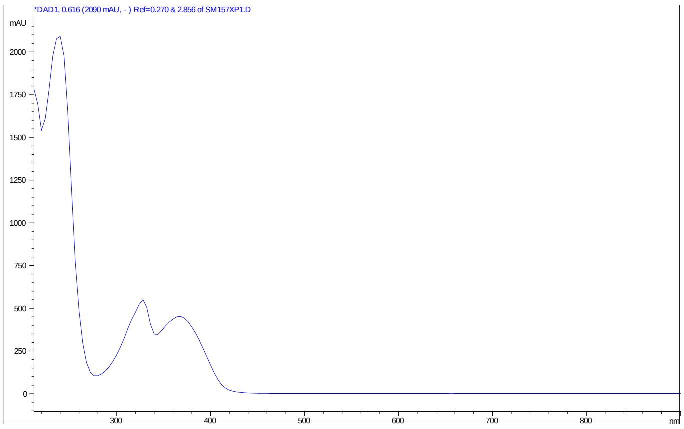

Figure S20. UV spectrum of compound **4**

ISCID=75 eV

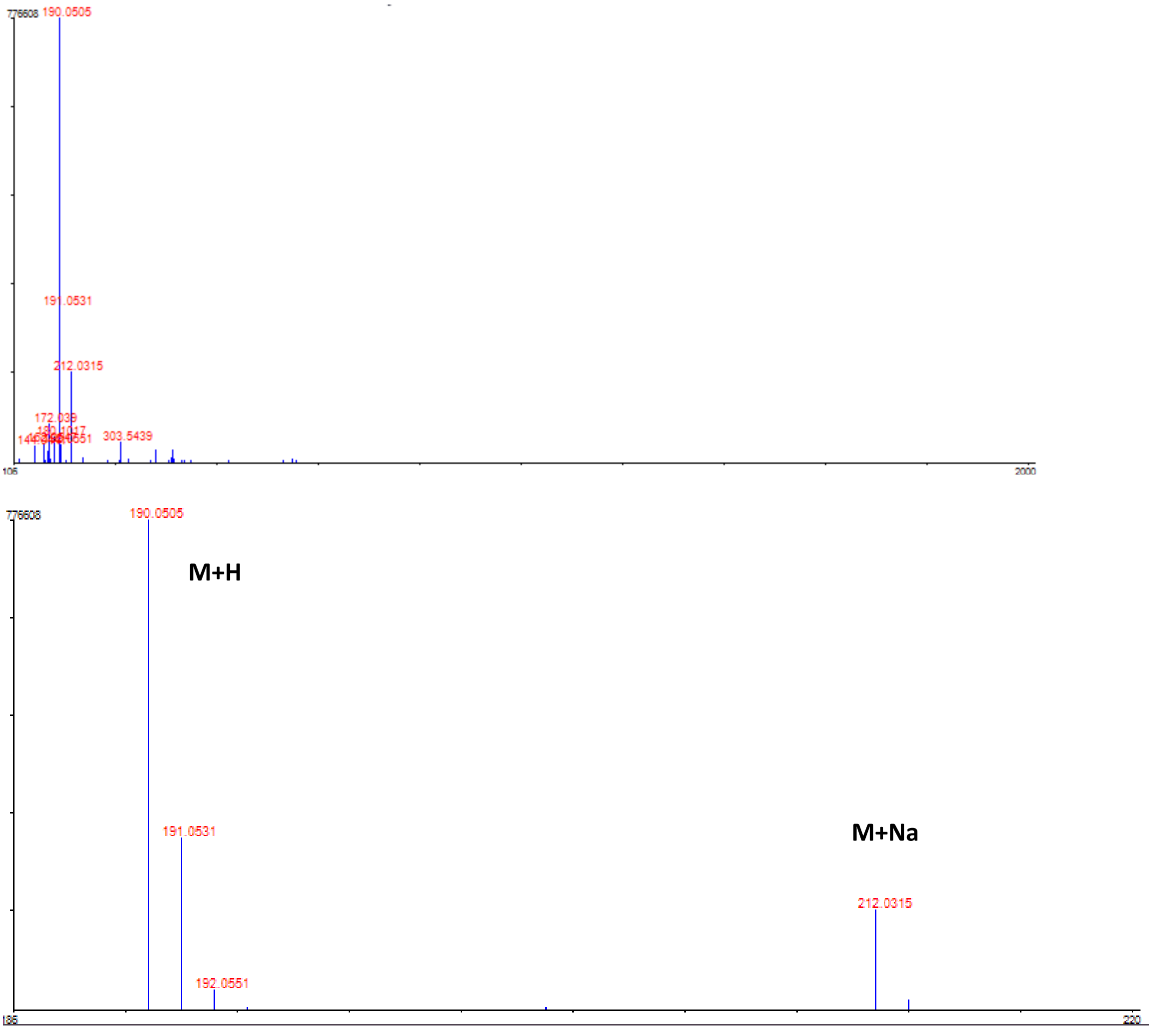

Figure S21. ESI TOF spectrum of compound **4**

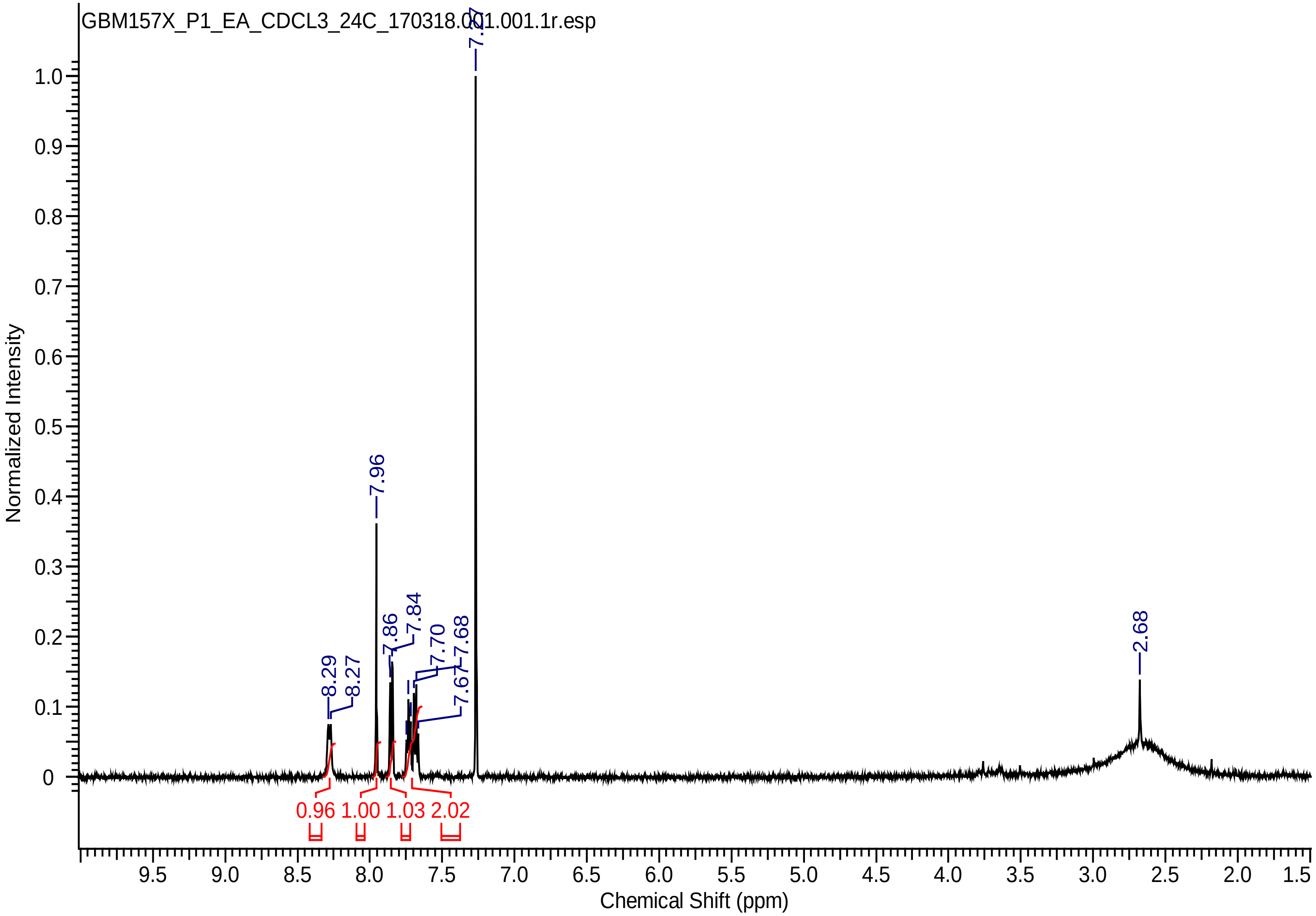

Figure S22. ^{1}H NMR ($CDCl_3$, 500 MHz) of compound **4**

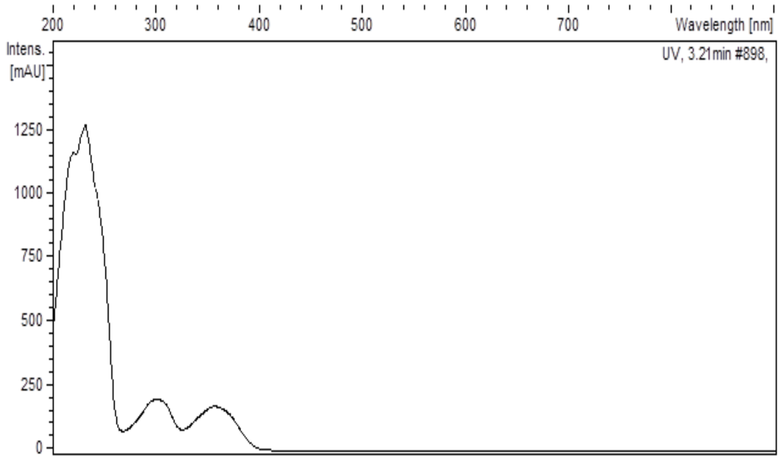

Figure S23. UV spectrum of compound **5**

ISCID=75 eV

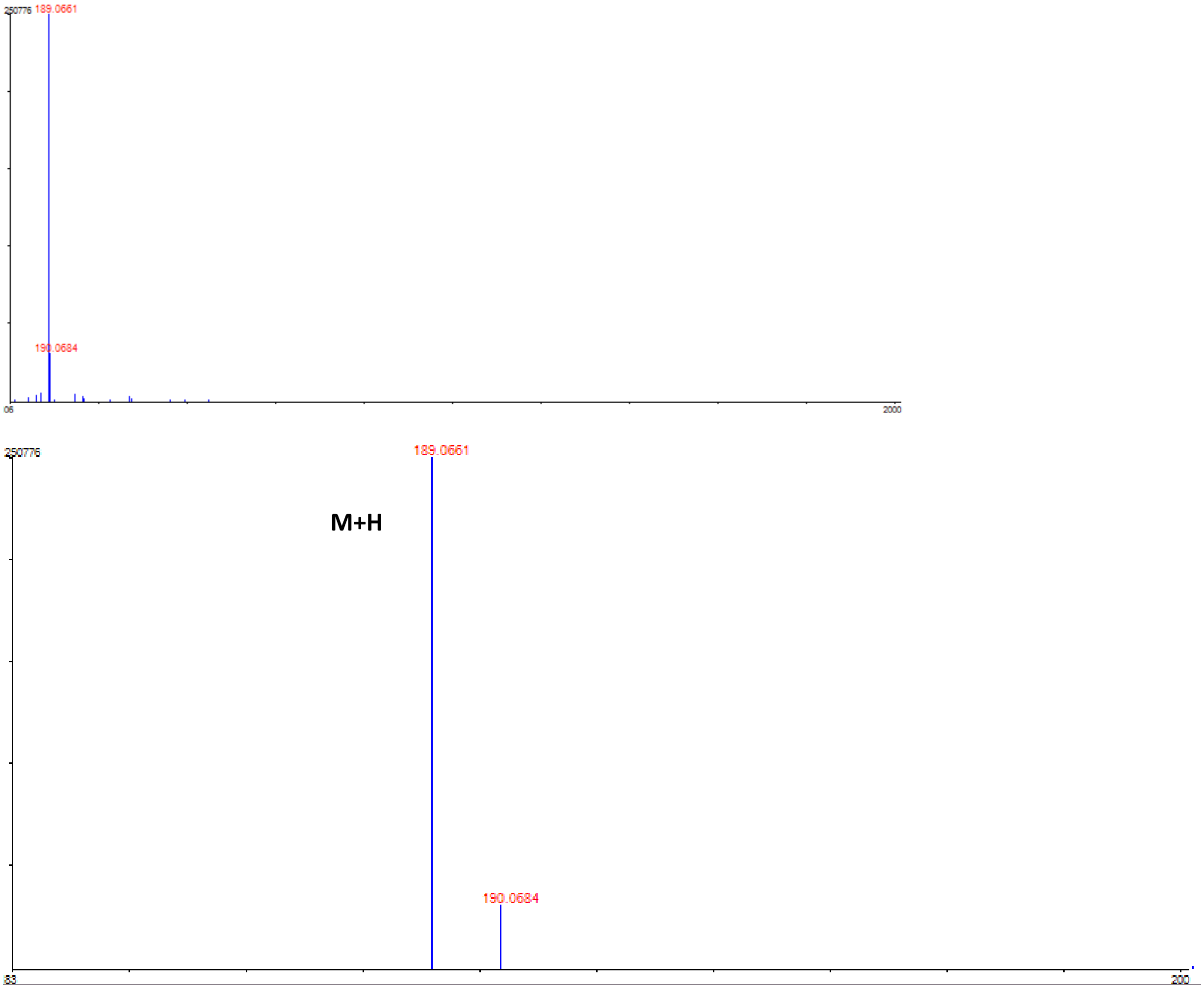

Figure S24. ESI TOF spectrum of compound **5**

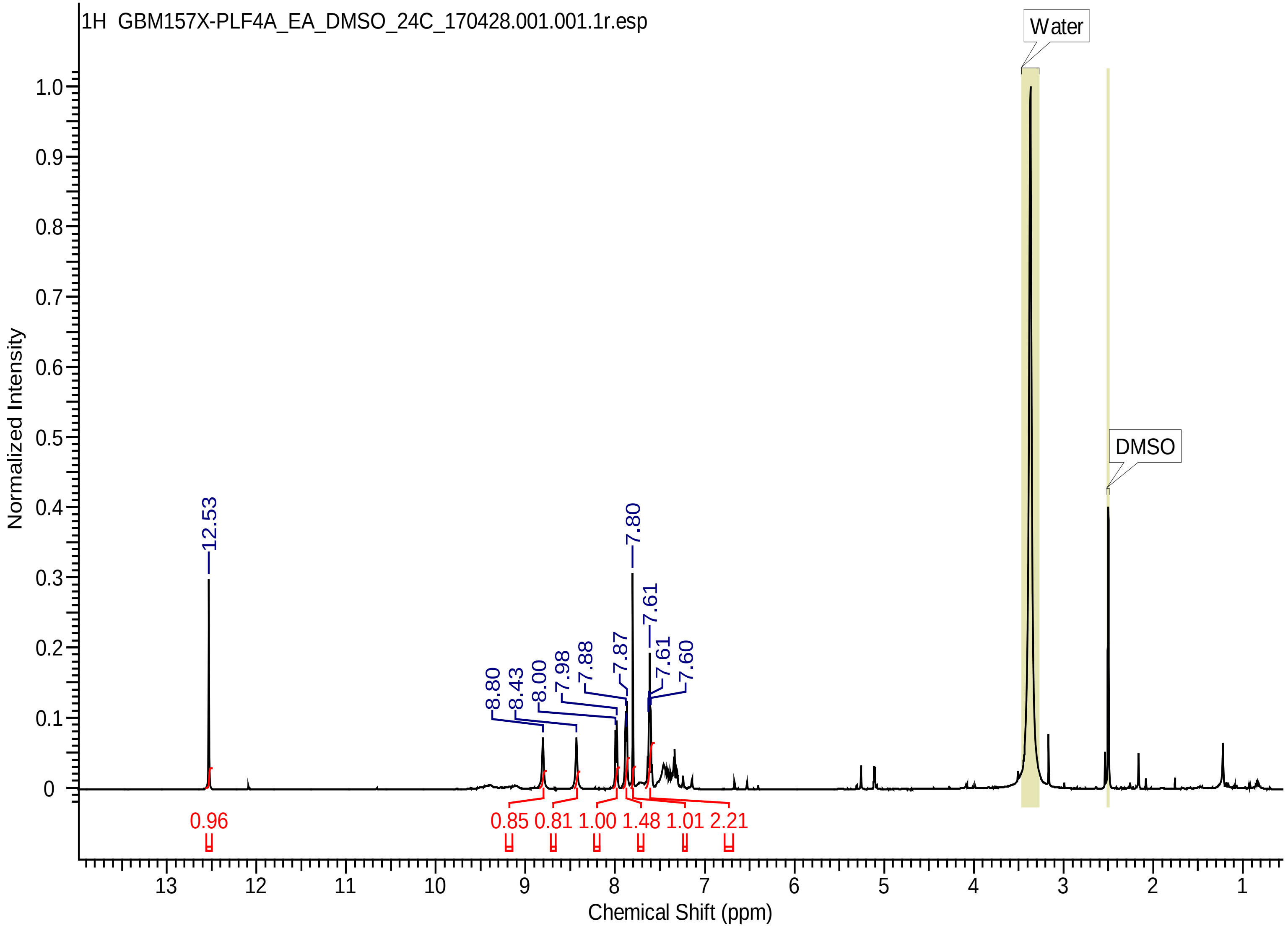

Figure S25. ^{1}H NMR (DMSO-*d6*, 500 MHz) of compound **5**

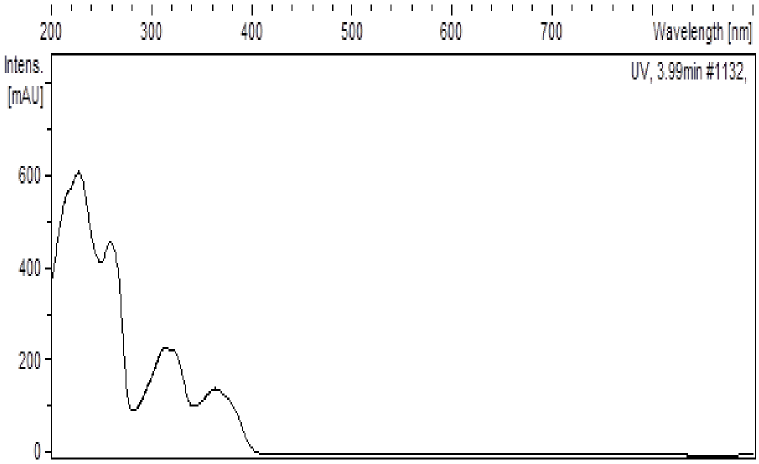

Figure S26. UV spectrum of compound **6**

ISCID=75 eV

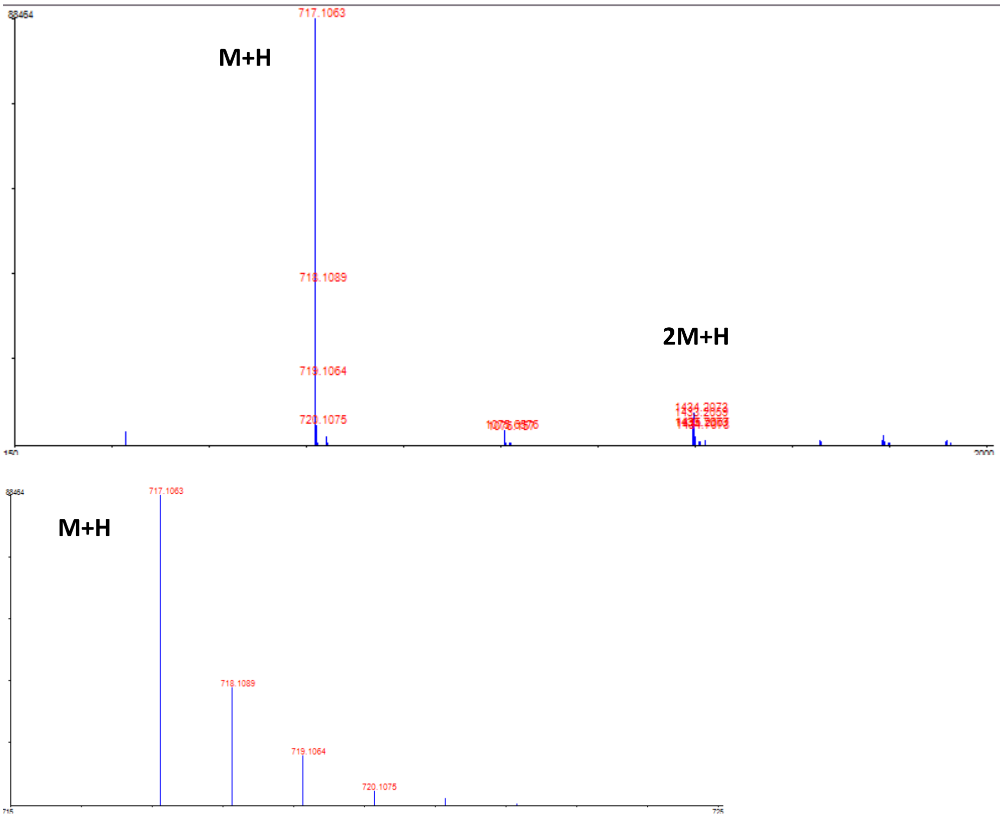

Figure S27. ESI TOF spectrum of compound **6**.

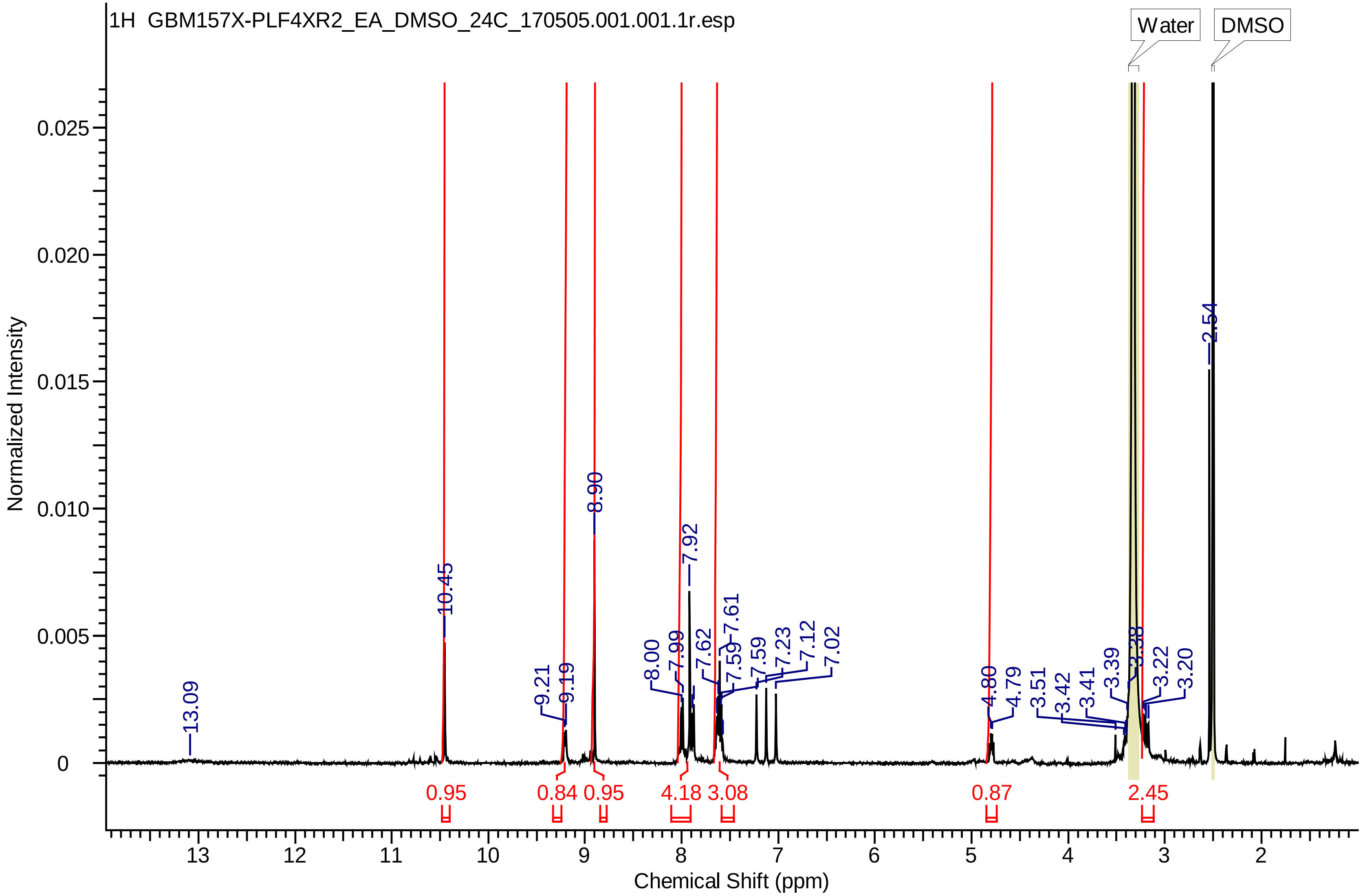

Figure S28. ¹H NMR (DMSO-*d6*, 500 MHz) of compound **6**.

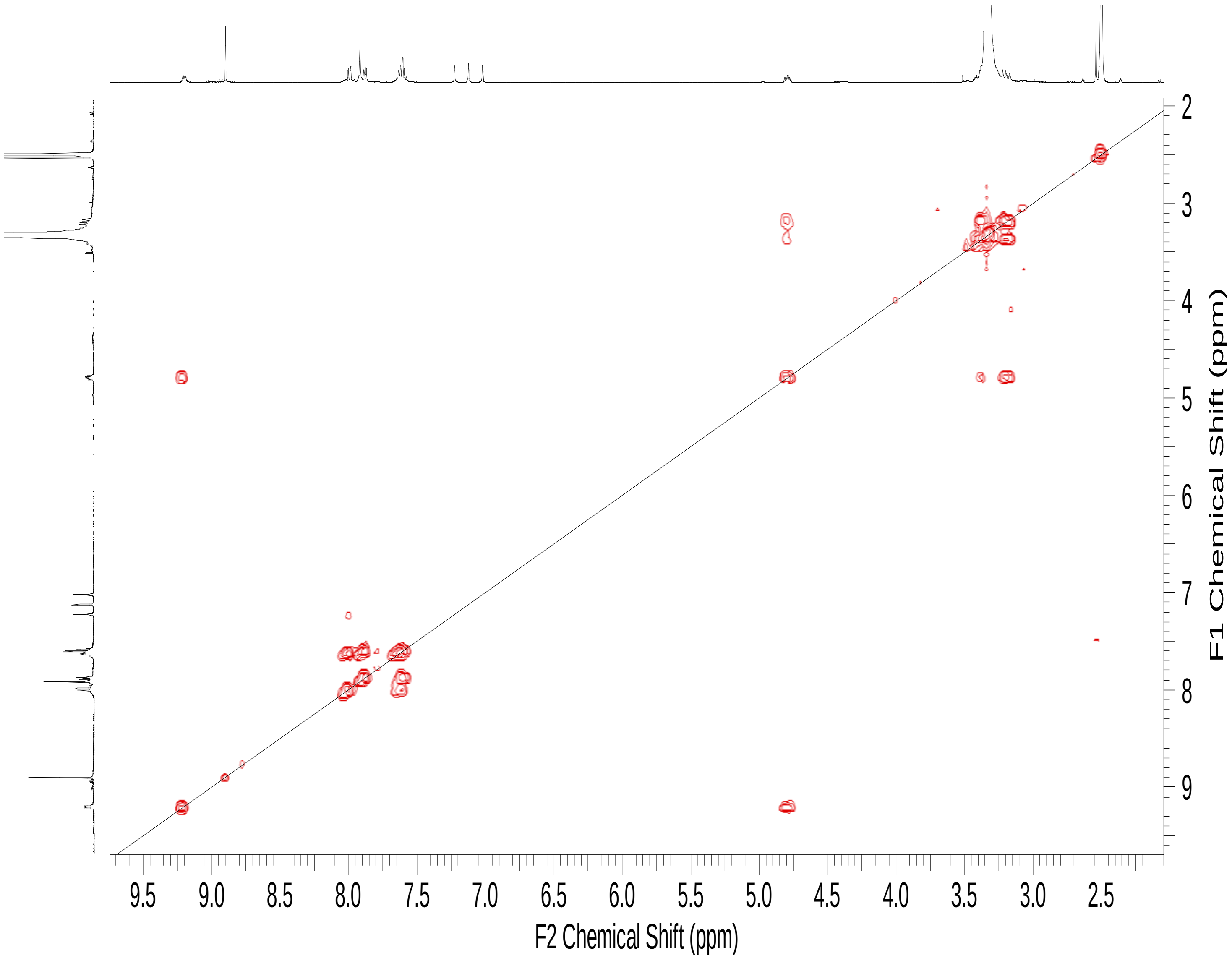

Figure S29. COSY spectrum of compound **6**.

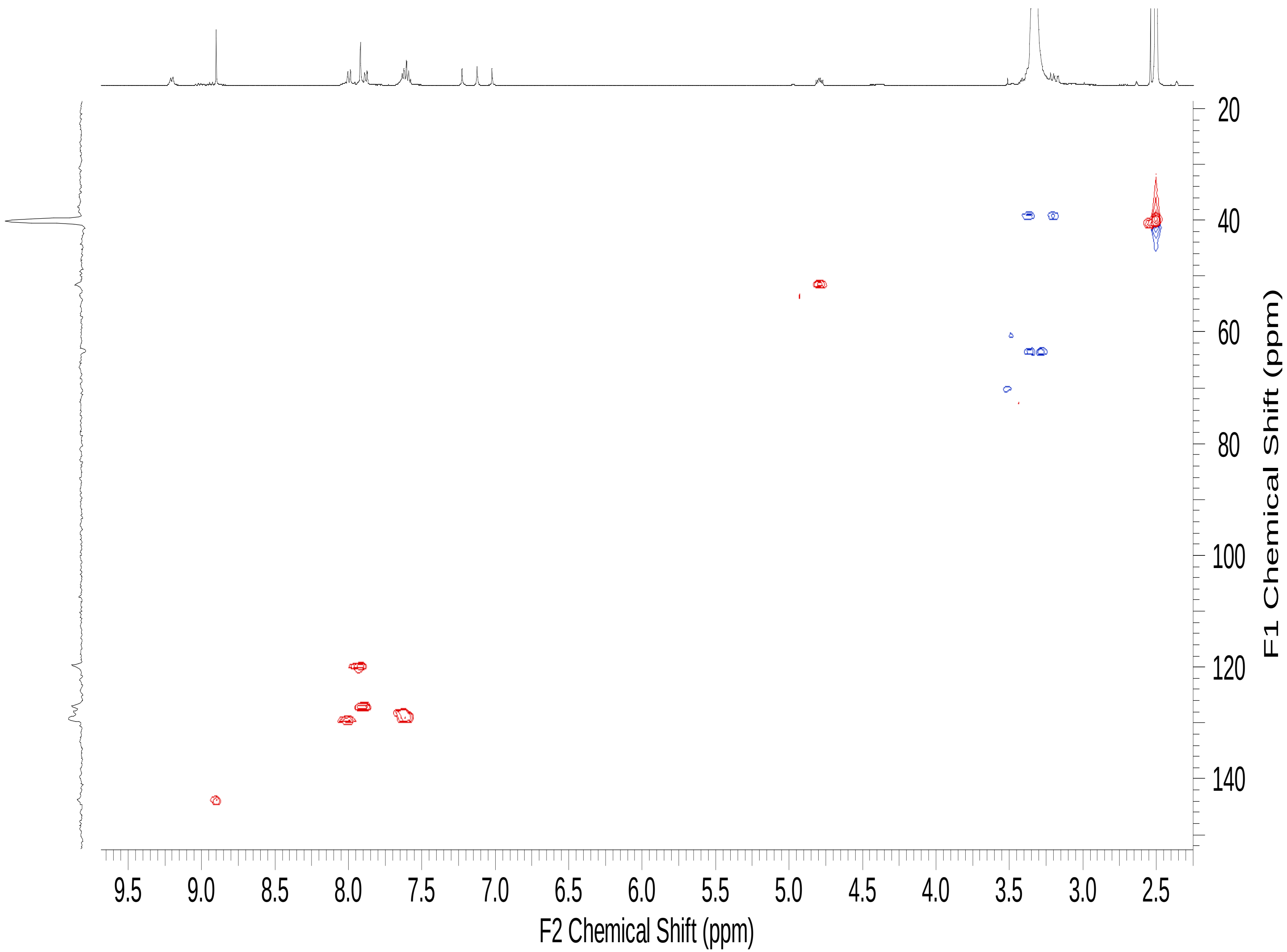

Figure S30. HSQC spectrum of compound **6**.

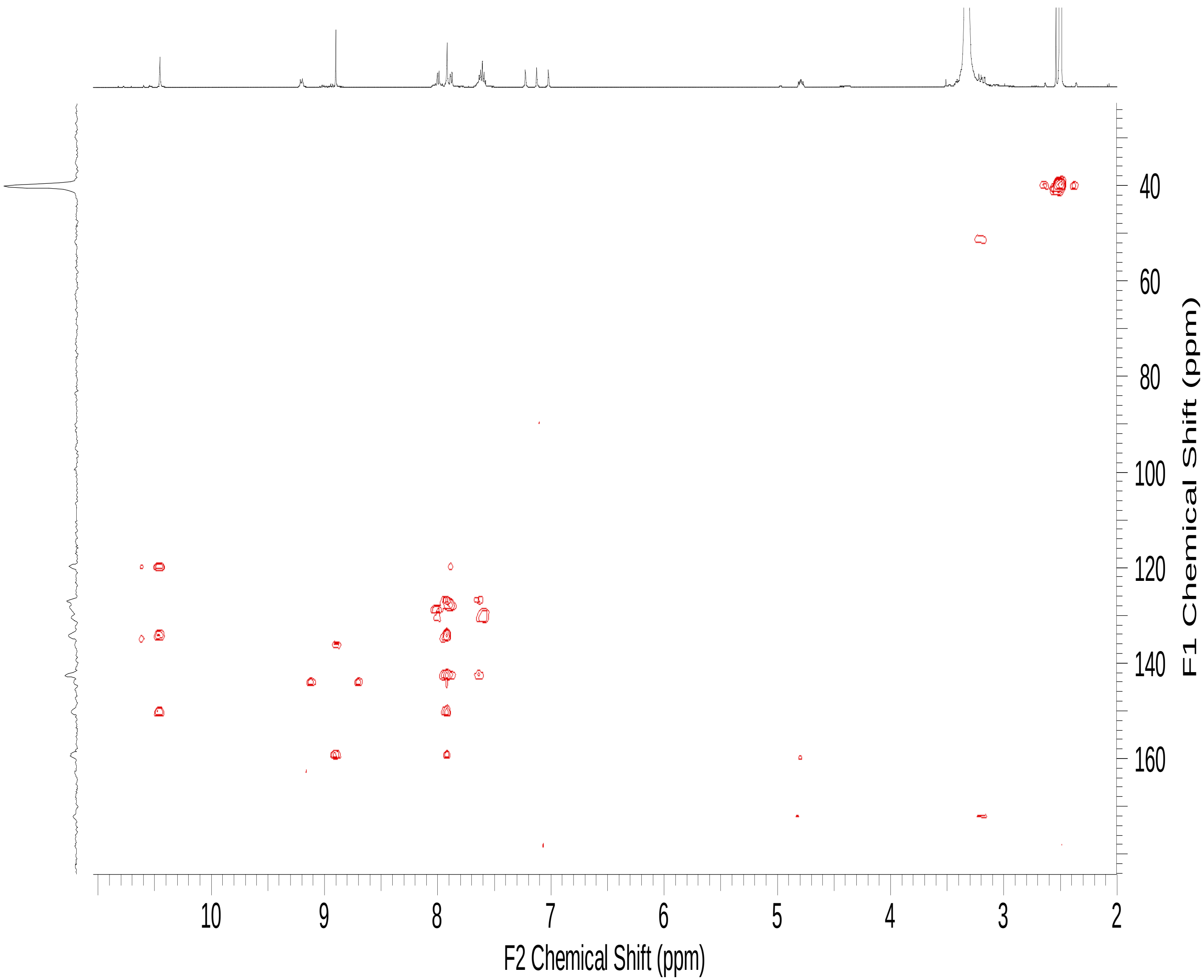

Figure S31. HMBC spectrum of compound **6**.

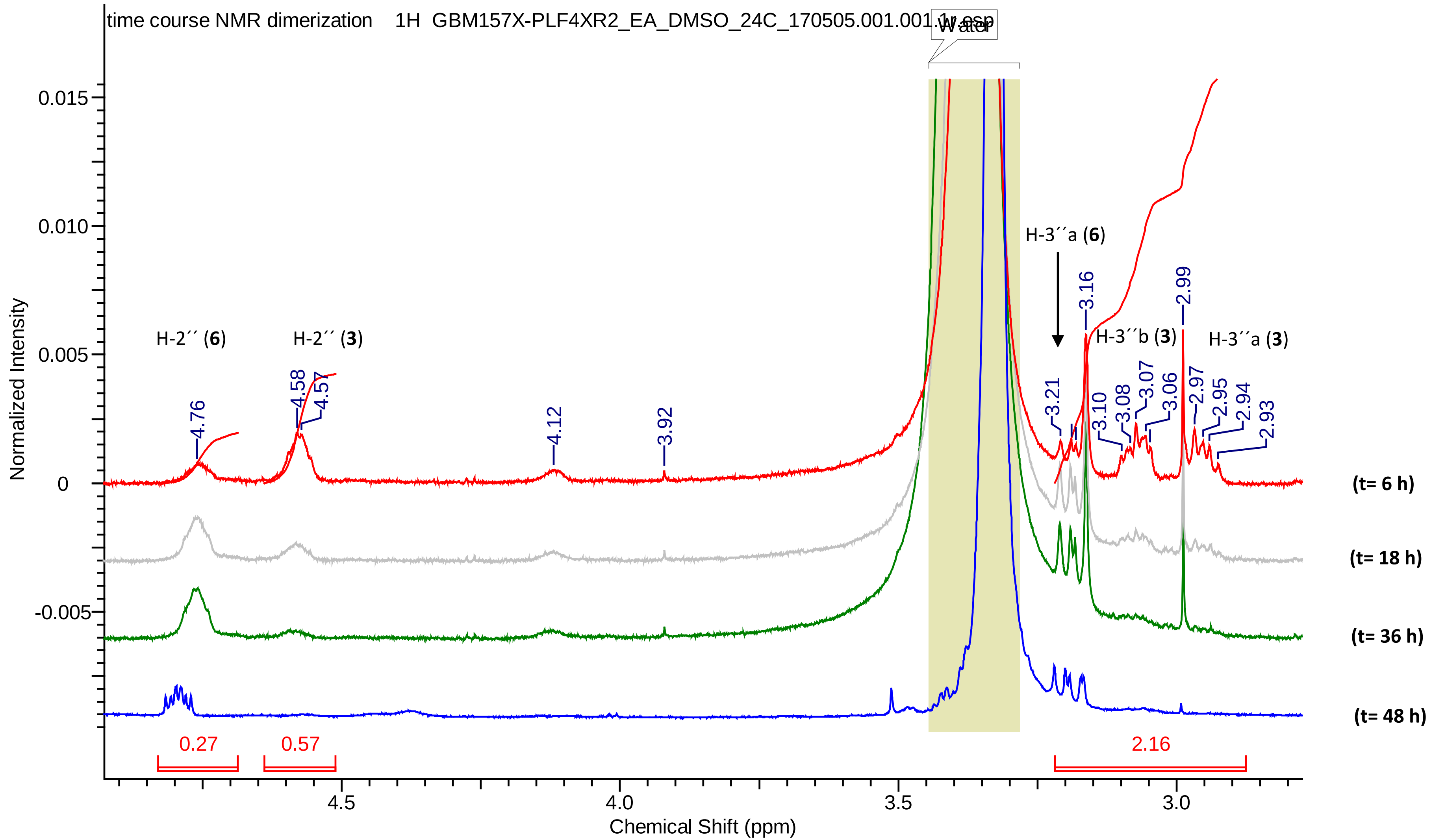

Figure S32. ^{1}H-NMR (DMSO-*d6*, 500 MHz) time-course conversion of **3** into **6**. Overlay of ^{1}H-NMR experiments (zoom).

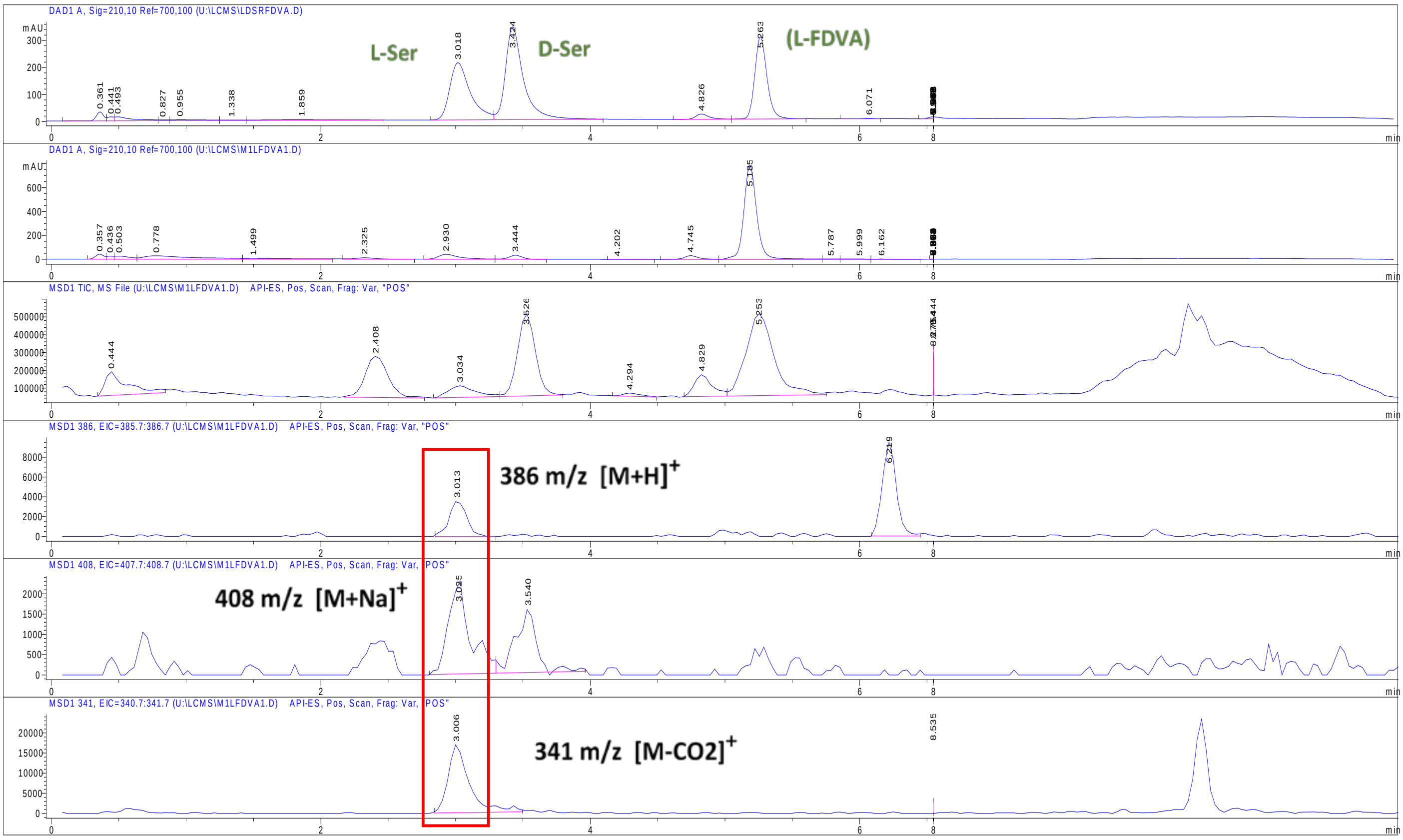

Fig. S33. HPLC traces of Marfey´s analysis of compound **1**

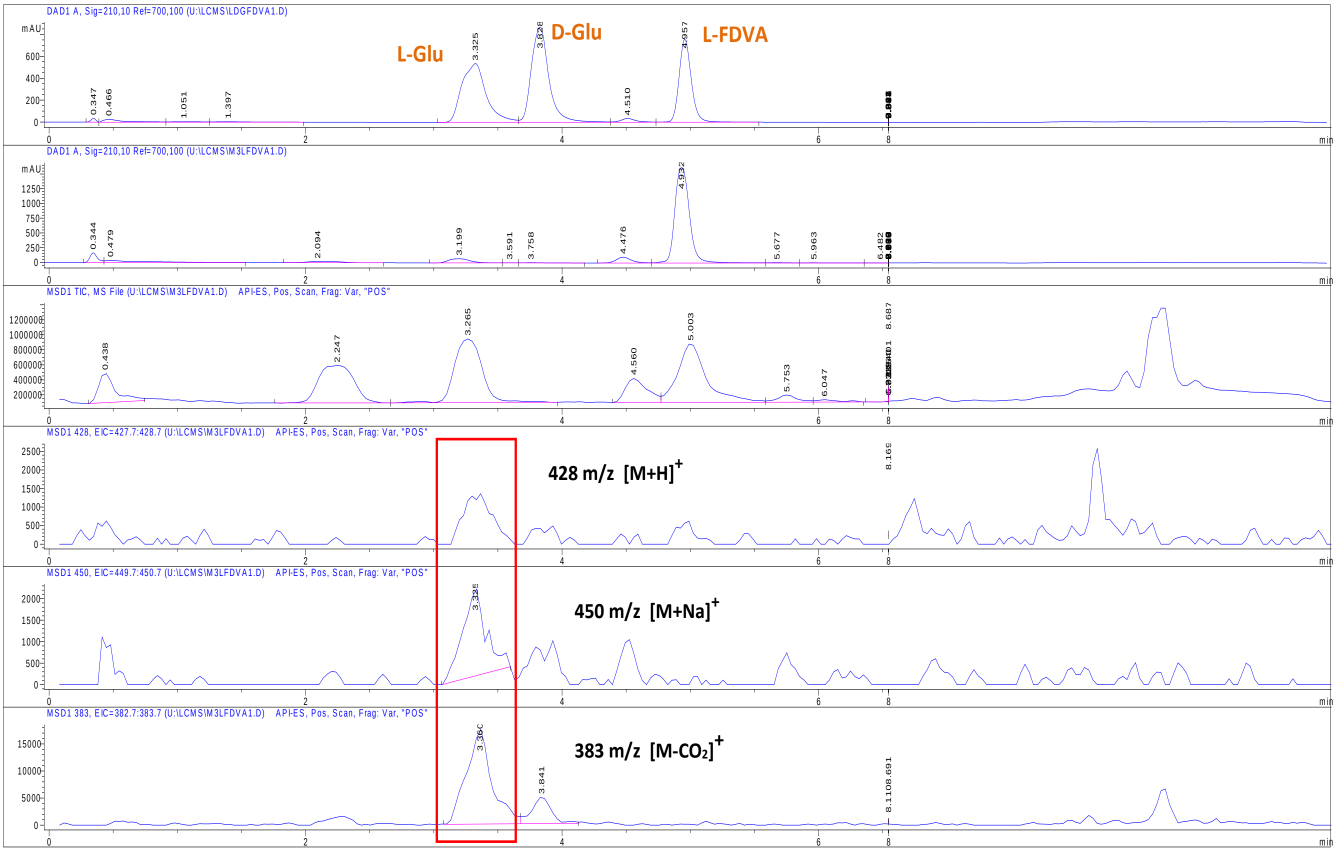

Fig. S34. HPLC traces of Marfey´s analysis of compound **2**

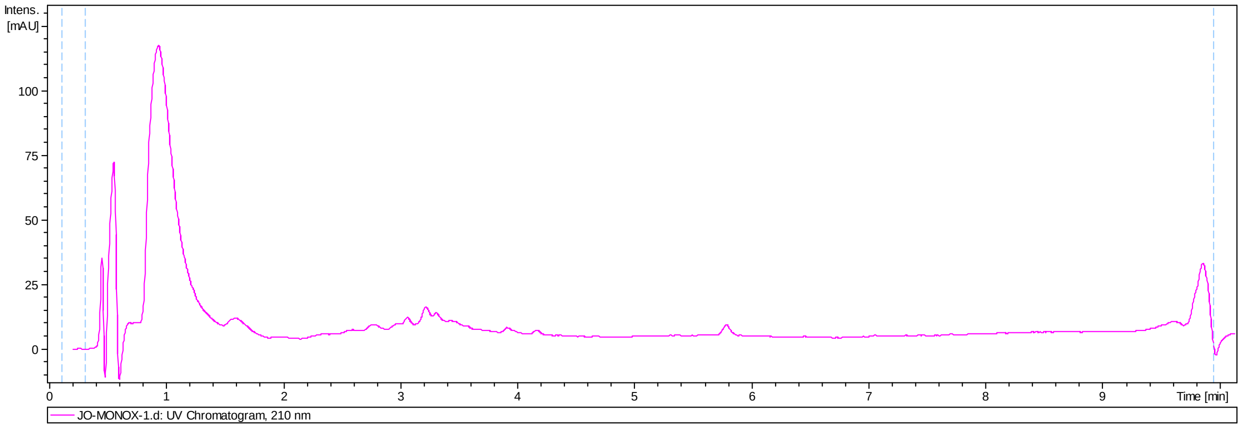

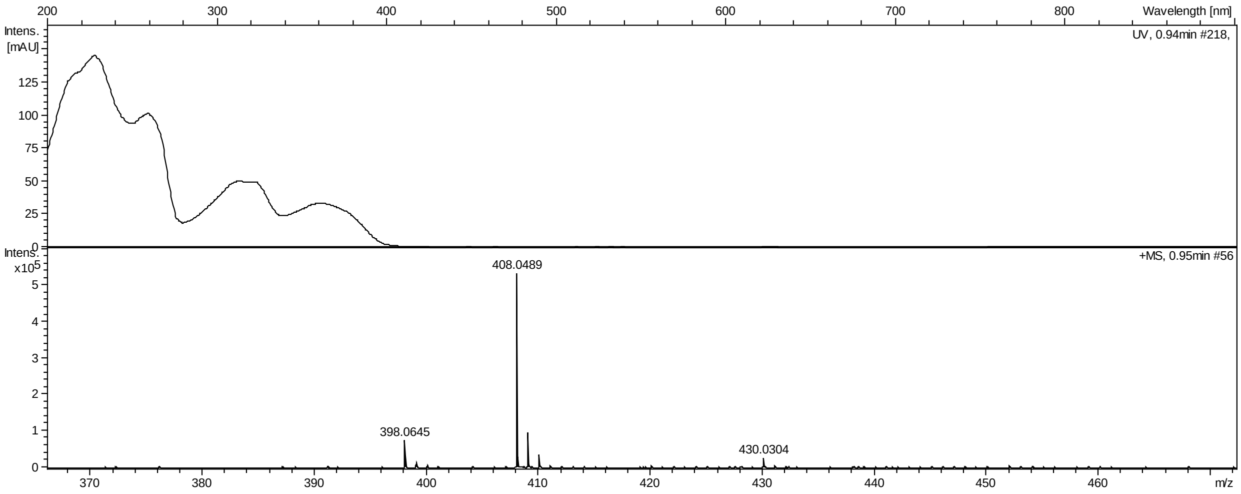

(zoom)

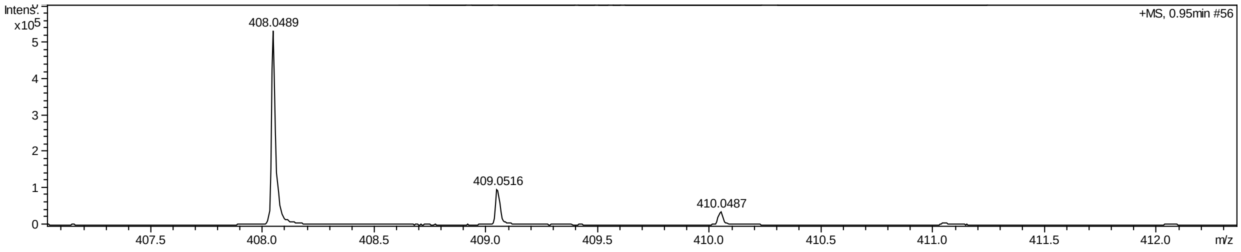

Fig. S35. LC-HRMS analysis of the oxidation crude of compound **3** (LC-HRMS chromatogram, UV spectrum and (+)-ESI-TOF spectrum of the oxidation product of **3**)

**Sample Name:**	**JO-MONOX-1**
**Sample ID:**	\\maXis01\D\Data\20180927\JO-MONOX-1.d
**Plate Pos:**	V09
**RT:**	0.95
**Area:**	19854148
**Intensity:**	1008983
**Signal To Noise:**	62965
**Suggested mass:**	407.0410
**Suggested formula:**	C16H13N3O8S
**Medina ID:**	MED-203610

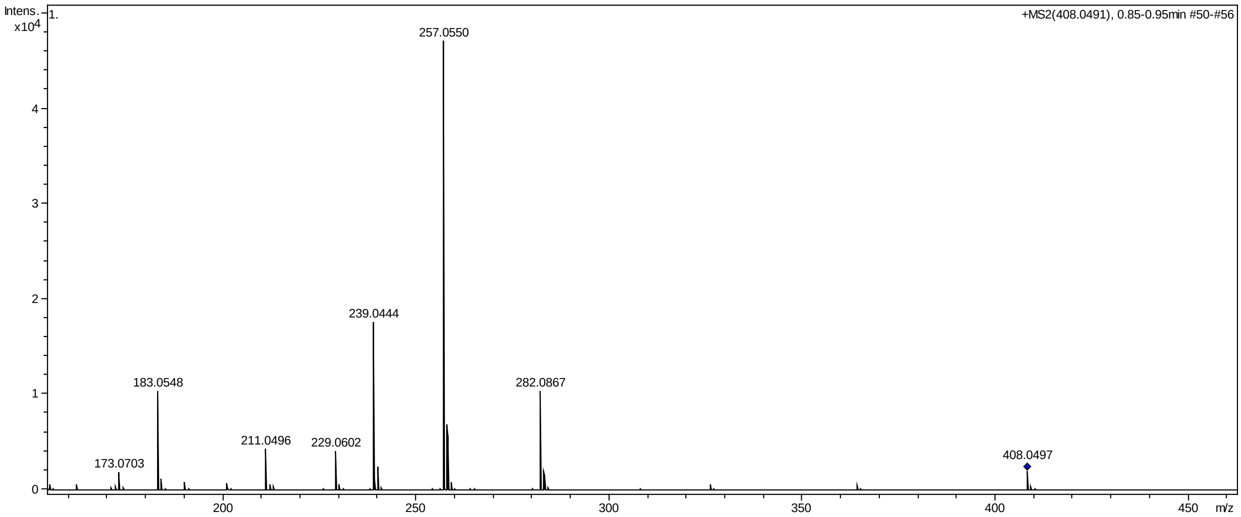

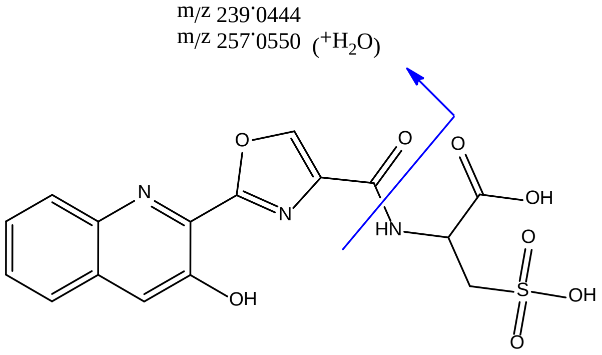

$C_{16}H_{13}N_3O_8S$

Fig. S36. HRMS-MS spectrum of the oxidation product of compound **3**

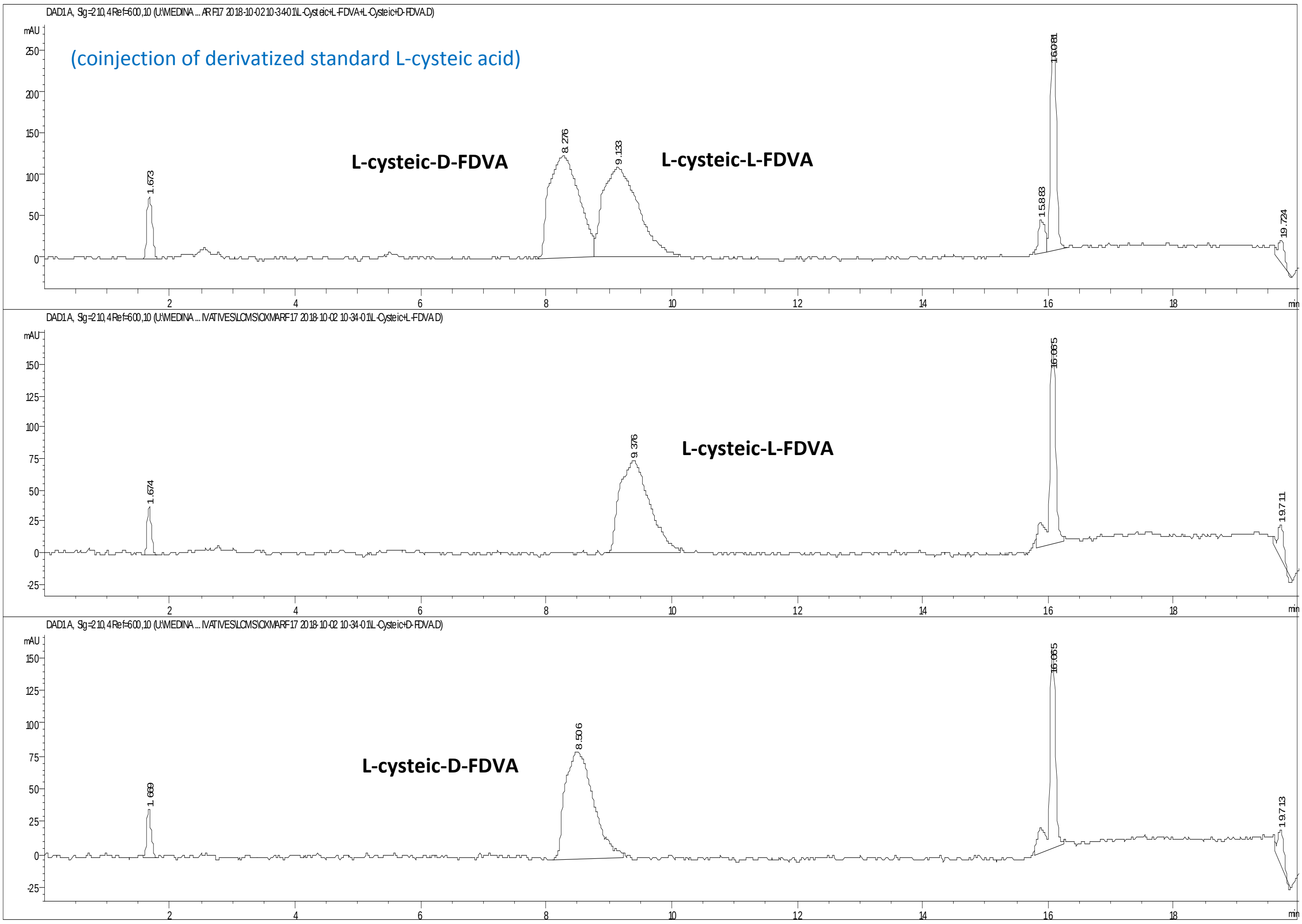

Fig. S37. HPLC traces of L- and D-FDVA derivatives of standard L-cysteic acid

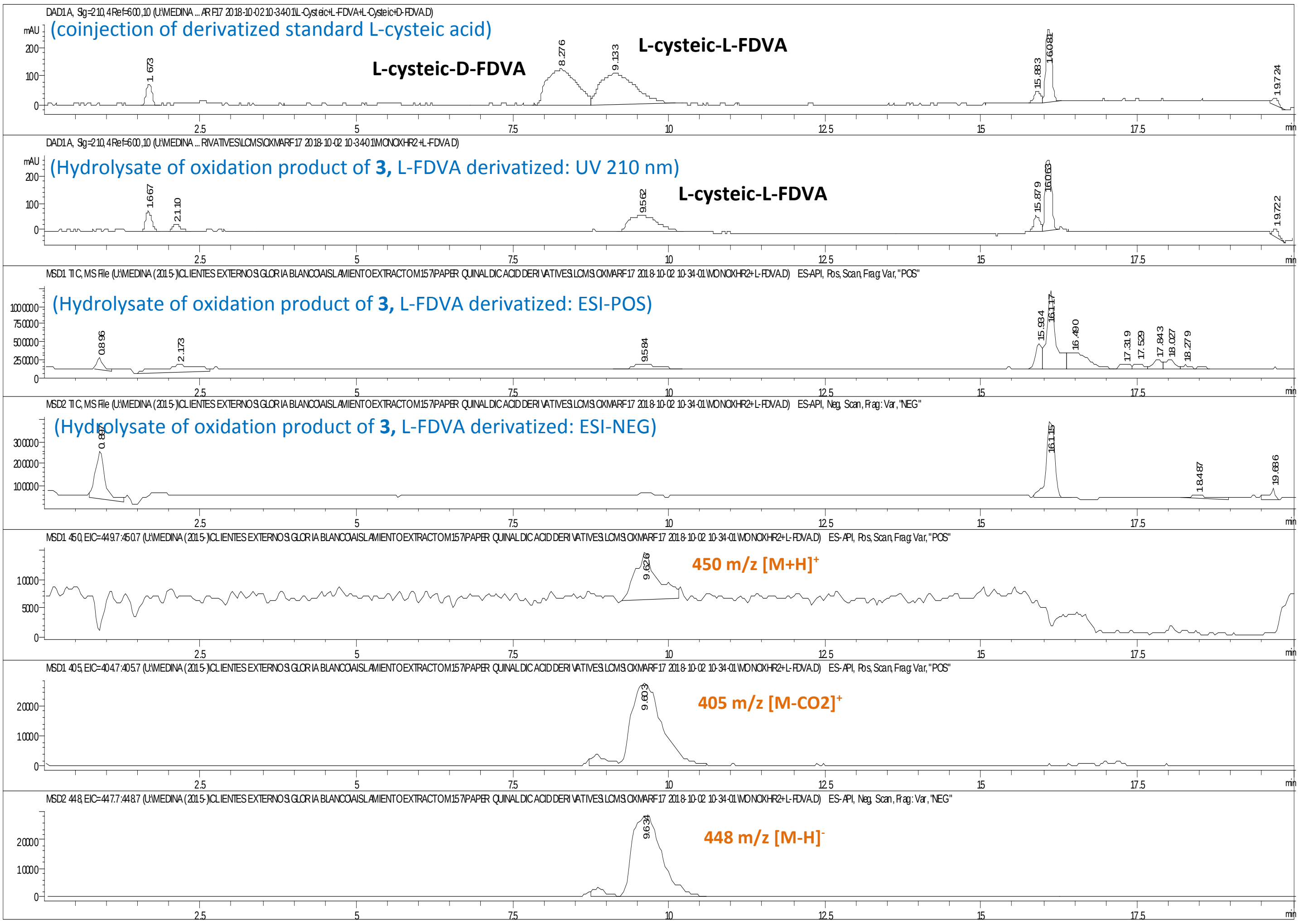

Fig. S38. HPLC traces of Marfey´s analysis of oxidation product of compound **3**
